# When is the allele-sharing dissimilarity between two populations exceeded by the allele-sharing dissimilarity of a population with itself?

**DOI:** 10.1515/sagmb-2023-0004

**Published:** 2023-12-12

**Authors:** Xiran Liu, Zarif Ahsan, Tarun K. Martheswaran, Noah A. Rosenberg

**Affiliations:** Institute for Computational and Mathematical Engineering, Stanford University, Stanford, CA 94305, USA; Department of Biology, Stanford University, Stanford, CA 94305, USA

**Keywords:** allele-sharing, genetic dissimilarity, population genetics

## Abstract

Allele-sharing statistics for a genetic locus measure the dissimilarity between two populations as a mean of the dissimilarity between random pairs of individuals, one from each population. Owing to within-population variation in genotype, allele-sharing dissimilarities can have the property that they have a nonzero value when computed between a population and itself. We consider the mathematical properties of allele-sharing dissimilarities in a pair of populations, treating the allele frequencies in the two populations parametrically. Examining two formulations of allele-sharing dissimilarity, we obtain the distributions of within-population and between-population dissimilarities for pairs of individuals. We then mathematically explore the scenarios in which, for certain allele-frequency distributions, the within-population dissimilarity – the mean dissimilarity between randomly chosen members of a population – can exceed the dissimilarity between two populations. Such scenarios assist in explaining observations in population-genetic data that members of a population can be empirically more genetically dissimilar from each other on average than they are from members of another population. For a population pair, however, the mathematical analysis finds that at least one of the two populations always possesses smaller within-population dissimilarity than the value of the between-population dissimilarity. We illustrate the mathematical results with an application to human population-genetic data.

## 1 Introduction

Statistics that measure the genetic dissimilarity between pairs of populations are widely used for interpreting population-genetic data (Bowcock et al. 1994; Chakraborty and Jin 1993; Gao and Martin 2009; Mountain and Cavalli-Sforza 1997; Mountain and Ramakrishnan 2005; Rosenberg 2011; Tal 2013; Witherspoon et al. 2007). Patterns in numerical values of the statistics appear in calculations of the relative similarity and dissimilarity of different human groups (Mountain and Ramakrishnan 2005; Rosenberg 2011; Witherspoon et al. 2007). Further, genetic dissimilarity statistics, often termed “genetic distances,” underlie frequently applied tools for data analysis and visualization, including methods such as evolutionary tree construction (Bowcock et al. 1994) and multidimensional scaling (Gao and Martin 2009).

Population-level genetic dissimilarity statistics computed at a single genetic locus often proceed by considering pairs of vectors, **p** and **q**, representing the allele frequencies of two populations. Each vector consists of nonnegative entries that sum to 1. Hence, for a locus with *I* distinct alleles, such a genetic dissimilarity statistic has domain Δ^*I*−1^ × Δ^*I*−1^, where Δ^*I*−1^ is the simplex $\left\{p_{1},p_{2},\ldots ,p_{I}:\sum_{i=1}^{I}p_{i}=1\;\text{and }p_{i}\geq 0\;\text{for all }i\right\}$ .

Among the many genetic dissimilarity statistics that are available (Jorde 1985; Nei 1987), those known as *allele-sharing dissimilarities* form a distinctive subset. Such statistics view a dissimilarity between two populations as the mean of a dissimilarity between pairs of individuals, one from one population and one from the other. With this perspective, they have a simple interpretation as a population-level generalization of an individual-level statistic. They also have a natural connection to a fundamental computation in human population genetics – the apportionment of genetic diversity among different levels of genetic structure (Edge et al. 2022; Lewontin 1972) – which can be viewed in terms of various mean pairwise dissimilarities across certain subsets of individuals (Rosenberg 2011).

Unlike most dissimilarity statistics – such as those based on such principles as the Euclidean distance between functions of allele frequency vectors (Cavalli-Sforza and Edwards 1967) or the dot product of these vectors (Nei 1972) – because they emerge from inter-individual computations among non-identical individuals, allele-sharing dissimilarities can produce nonzero values for the dissimilarity between a polymorphic population and itself. This feature assists in understanding a property of genetic variation in structured populations: the extent to which genetic dissimilarity of individuals from the same population ever exceeds genetic dissimilarity of individuals from different populations, if at all.

Because individuals in a population generally possess a larger number of recent shared ancestors than individuals from different populations, a perspective focused on population-genetic descent predicts that individuals from the same population will be genetically more similar than individuals from different populations. Indeed, in human population genetics, studies of allele-sharing dissimilarity find that the mean dissimilarity across pairs of individuals from different populations does exceed the mean dissimilarity for pairs from the same populations (Mountain and Ramakrishnan 2005; Rosenberg 2011; Tal 2013; Witherspoon et al. 2007). However, such studies also find a perhaps unexpected result that the allele-sharing dissimilarity for *some* pairs of individuals from the same population can exceed the dissimilarity for *some* pairs from different populations.

Here, we seek to explain the properties of allele-sharing dissimilarities within and between populations. We study mathematical properties of population-level allele-sharing dissimilarities under the assumption that individuals in a population represent random draws from the vector of allele frequencies in the population. We consider mean allele-sharing dissimilarities for pairs of individuals from the same population and for pairs of individuals from different populations, evaluating the conditions on allele-frequency vectors under which the allele-sharing dissimilarity for a population to itself can exceed the allele-sharing dissimilarity between two populations. We interpret the results in relation to ongoing efforts to understand human genetic similarity and difference.

## 2 Methods

### 2.1 Allele-sharing dissimilarities

An allele-sharing dissimilarity (ASD) is a type of dissimilarity that is based on counting the number of alleles shared at a locus between two diploid individuals. We consider two different versions of the ASD concept.

In one ASD variant, which we denote by $D_{1}$ , “allele-sharing” for two diploid individuals is interpreted as the number of shared elements in their multisets of alleles. Consider a locus with four distinct alleles, the minimum number required so that all possible cases exist. Call these alleles *A*, *B*, *C*, and *D*. For $D_{1}$ , two individuals both with genotype *AB* have 2 alleles shared, as the sets {*A*, *B*} and {*A*, *B*} have 2 identical elements. An individual with genotype *AB* and an individual with genotype *AC* have 1 allele shared, as the sets {*A*, *B*} and {*A*, *C*} have 1 element shared between them, namely *A*. Two individuals with genotype *AA* have 2 alleles shared, as multisets {*A*, *A*} and {*A*, *A*} have 2 shared elements, *A* and *A*. The dissimilarity $D_{1}$ then uses 1 minus half the number of the shared alleles as the dissimilarity; the normalization ensures that $D_{1}$ lies in [0,1] (Gao and Martin 2009; Mountain and Cavalli-Sforza 1997). With 0, 1, and 2 shared alleles, the dissimilarity equals 1, $\frac{1}{2}$ , and 0, respectively.

Another variant of ASD, which we denote by $D_{2}$ , instead considers alleles individually, evaluating the fraction of pairs of alleles, one from the first individual and one from the second, that are distinct (Mountain and Ramakrishnan 2005). For two individuals with genotype *AB*, $D_{2}$ is equal to $\frac{1}{2}$ , because among the four possible pairs of alleles – (*A*, *A*), (*A*, *B*), (*B*, *A*), and (*B*, *B*), where the first entry in the pair represents an allele from the first individual and the second entry is an allele from the second individual – two of four contain distinct alleles.

Table 1 shows all seven possible pairs of unordered diploid genotypes for two individuals and their corresponding dissimilarities measured by $D_{1}$ and $D_{2}$ . In only two of seven cases do the two dissimilarities differ.

**Table 1:** Two variants of allele-sharing dissimilarity. All possible pairs of unordered genotypes are shown, along with their values of $D_{1}$ and $D_{2}$ .

Case	Genotypes	$D_{1}$	$D_{2}$
1	*AA*, *AA*	0	0
2	*AA*, *AB*	$\frac{1}{2}$	$\frac{1}{2}$
3	*AA*, *BB*	1	1
4	*AA*, *BC*	1	1
5	*AB*, *AB*	0	$\frac{1}{2}$
6	*AB*, *AC*	$\frac{1}{2}$	$\frac{3}{4}$
7	*AB*, *CD*	1	1

### 2.2 Notation

Consider a locus with *I* distinct alleles. We consider allele-frequency vectors in each of two populations. In Population 1, the allele frequencies are **p** = (*p*_1_, *p*_2_, …, *p*_*I*_), where *p*_*i*_ represents the frequency of allele *i*. In Population 2, they are **q** = (*q*_1_, *q*_2_, …, *q*_*I*_). The frequencies satisfy 0 ≤ *p*_*i*_, *q*_*i*_ ≤ 1 for all *i*, and $\sum_{i=1}^{I}p_{i}=\sum_{i=1}^{I}q_{i}=1$ .

We are interested in mathematical properties of the distribution of ASD measure D , for pairs of populations – possibly the same population – where D can refer to $D_{1}$ or $D_{2}$ . We denote the dissimilarity D between two randomly chosen individuals within the same population with allele-frequency vector **p** by $D^{w}\left(p\right)$ , and the corresponding dissimilarity between two randomly chosen individuals from different populations with allele-frequency vectors **p** and **q** by $D^{b}\left(p,q\right)$ . We often drop the arguments for convenience.

We will have occasion to use various symmetric sums involving allele frequencies. For *t* = 1, 2, 3, 4, for expressions in the separate populations, we use the notation

(1) $$\sigma_{t}=\overset{I}{\underset{i=1}{\sum }}p_{i}^{t},\;\tau_{t}=\overset{I}{\underset{i=1}{\sum }}q_{i}^{t},$$

where *σ*_1_ = *τ*_1_ = 1.

For expressions involving both populations, we use

(2) $$\rho_{tu}=\overset{I}{\underset{i=1}{\sum }}p_{i}^{t}q_{i}^{u},$$

where (*t*, *u*) is equal to (1,1), (1,2), (2,1), or (2,2). Note that each of these sums can be viewed as an inner product.

### 2.3 Assumptions

We seek to perform ASD computations under the assumption that individuals are sampled at random from allele-frequency distributions. With this perspective, for a random pair of individuals, an ASD measure is a random variable that depends on the allele-frequency vectors of two populations of interest, treated as parameters.

At a given locus, we assume that the two alleles of an individual are sampled independently, so that diploid genotypes in a population are assumed to follow Hardy–Weinberg proportions. In other words, the probabilities of diploid genotypes in a population with allele-frequency vector **p** equal $p_{i}^{2}$ for homozygous genotypes and 2*p*_*i*_ *p*_*j*_ for heterozygous unordered genotypes, with *i* ≠ *j*.

## Distribution of $D^{w}$

3

We first compute allele-sharing dissimilarities between random pairs of individuals sampled from the same population, evaluating the properties of random variables $D_{1}^{w}$ and $D_{2}^{w}$ .

### Distribution of $D_{1}^{w}$

3.1

$D_{1}^{w}$ is a random variable that takes on values 0, $\frac{1}{2}$ , and 1. We compute its probability distribution, and we then evaluate its mean and variance.

$P\left[D_{1}^{w}=d\right]$ . We obtain the probability for each possible genotype combination in Table 1. These probabilities appear in Table 2, both as sums and as simplified polynomials.

**Table 2:** Probabilities of genotype combinations for pairs of individuals sampled from the same population. For each case, the probability is written as a sum, which is then simplified using Eq. (1).

Case	Genotypes	Probability	Simplified probability
1	*AA*, *AA*	$\overset{I}{\underset{i=1}{\sum }}p_{i}^{4}$	*σ*_4_
2	*AA*, *AB*	$4\sum_{i=1}^{I}p_{i}^{3}\sum_{\begin{array}{r} j=1\\ j\neq i \end{array}}^{I}p_{j}$	4*σ*_3_ − 4*σ*_4_
3	*AA*, *BB*	$\sum_{i=1}^{I}p_{i}^{2}\sum_{\begin{array}{r} j=1\\ j\neq i \end{array}}^{I}p_{j}^{2}$	$\sigma_{2}^{2}-\sigma_{4}$
4	*AA*, *BC*	$2\sum_{i=1}^{I}p_{i}^{2}\sum_{\begin{array}{r} j=1\\ j\neq i \end{array}}^{I}p_{j}\sum_{\begin{array}{r} k=1\\ k\neq i,j \end{array}}^{I}p_{k}$	$2\sigma_{2}-4\sigma_{3}-2\sigma_{2}^{2}+4\sigma_{4}$
5	*AB*, *AB*	$2\sum_{i=1}^{I}p_{i}^{2}\sum_{\begin{array}{r} j=1\\ j\neq i \end{array}}^{I}p_{j}^{2}$	$2{\sigma_{2}}^{2}-2\sigma_{4}$
6	*AB*, *AC*	$4\sum_{i=1}^{I}p_{i}^{2}\sum_{\begin{array}{r} j=1\\ j\neq i \end{array}}^{I}p_{j}\sum_{\begin{array}{r} k=1\\ k\neq i,j \end{array}}^{I}p_{k}$	$4\sigma_{2}-8\sigma_{3}-4\sigma_{2}^{2}+8\sigma_{4}$
7	*AB*, *CD*	$\sum_{i=1}^{I}p_{i}\sum_{\begin{array}{r} j=1\\ j\neq i \end{array}}^{I}p_{j}\sum_{\begin{array}{r} k=1\\ k\neq i,j \end{array}}^{I}p_{k}\sum_{\begin{array}{r} \ell =1\\ \ell \neq i,j,k \end{array}}^{I}p_{\ell }$	$1-6\sigma_{2}+8\sigma_{3}+3\sigma_{2}^{2}-6\sigma_{4}$

With the probabilities of all genotype combinations obtained, we can sum across genotype combinations to compute probabilities for $D_{1}^{w}\left(p\right)$ to equal 0, $\frac{1}{2}$ , and 1. The resulting probabilities appear in Table 3.

**Table 3:** Probability distribution of $D_{1}^{w}\left(p\right)$ , the allele-sharing dissimilarity $D_{1}^{w}$ for a pair of individuals sampled at random from a population with allele-frequency vector **p**. The table is obtained by summing entries in Table 2.

Value of the dissimilarity (*d*)	$P\left[D_{1}^{w}\left(p\right)=d\right]$
0	$2\sigma_{2}^{2}-\sigma_{4}$
$\frac{1}{2}$	$4\sigma_{2}-4\sigma_{3}-4\sigma_{2}^{2}+4\sigma_{4}$
1	$1-4\sigma_{2}+4\sigma_{3}+2\sigma_{2}^{2}-3\sigma_{4}$

$E\left[D_{1}^{w}\right]$ . The expected value of $D_{1}^{w}\left(p\right)$ can be computed from the full probability distribution, via

$$E\left[D_{1}^{w}\left(p\right)\right]=\sum_{d\in \left\{0,\frac{1}{2},1\right\}}dP\left[D_{1}^{w}\left(p\right)=d\right].$$

Using the probabilities in Table 3, the result is

(3) $$E\left[D_{1}^{w}\left(p\right)\right]=1-2\sigma_{2}+2\sigma_{3}-\sigma_{4}.$$

In the *I* = 2 case, using *p*_2_ = 1 − *p*_1_ so that $\sigma_{t}=p_{1}^{t}+{\left(1-p_{1}\right)}^{t}$ , Eq. (3) becomes:

(4) $$E\left[D_{1}^{w}\left(p\right)\right]=2p_{1}-4p_{1}^{2}+4p_{1}^{3}-2p_{1}^{4}.$$

**Figure 1:**
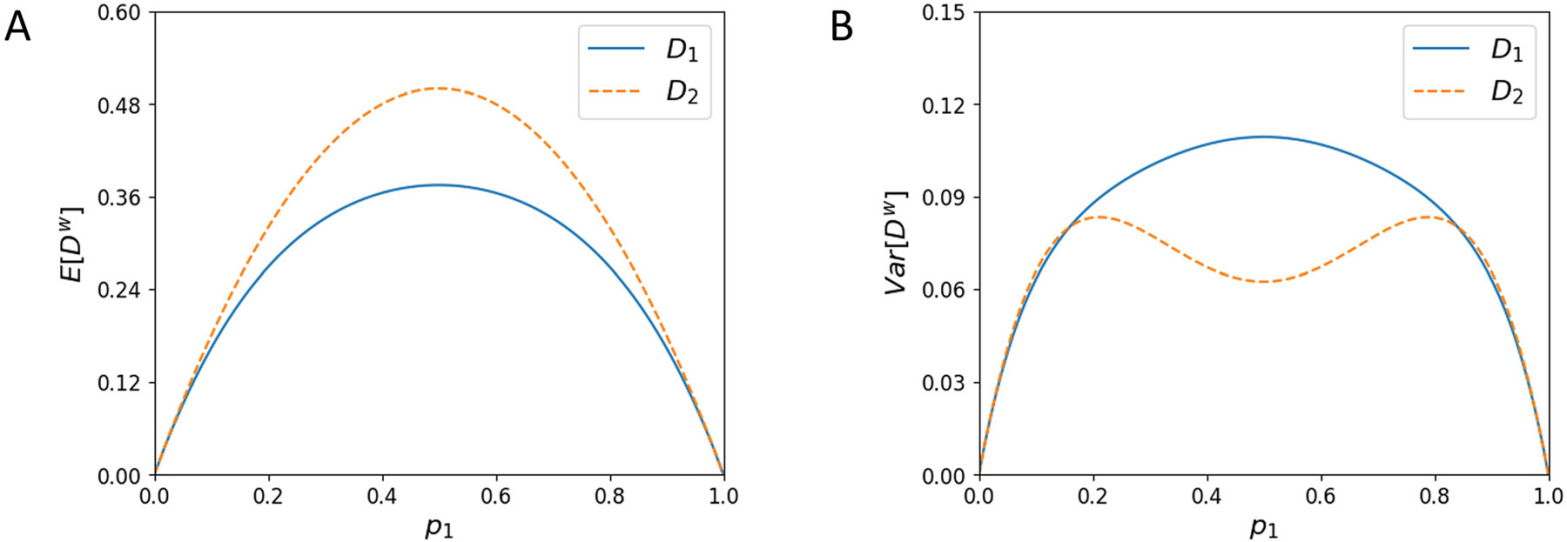
Mean and variance of the within-population dissimilarities $D_{1}^{w}$ and $D_{2}^{w}$ for *I* = 2 alleles as functions of the frequency *p*_1_ of one of the alleles. (A) Mean, Eqs. (4) and (10). (B) Variance, Eqs. (8) and (14).

Figure 1A plots Eq. (4) as a function of *p*_1_. In the figure, we can observe that the mean value of the dissimilarity increases from a value of 0 at *p*_1_ = 0, when the population is monomorphic, to a peak of $\frac{3}{8}$ at $p_{1}=\frac{1}{2}$ . It then decreases symmetrically to 0 at *p*_1_ = 1.

$Var\left[D_{1}^{w}\right]$ . To obtain the variance of the distribution of $D_{1}^{w}\left(p\right)$ , we first calculate

(5) $$\begin{array}{ll} E\left[D_{1}^{w}{\left(p\right)}^{2}\right] & =\sum_{d\in \left\{0,\frac{1}{2},1\right\}}{\text{d}}^{2}P\left[D_{1}^{w}\left(p\right)=d\right]\\ & =1-3\sigma_{2}+3\sigma_{3}+\sigma_{2}^{2}-2\sigma_{4}. \end{array}$$

The variance can then be obtained from Eqs. (3) and (5) by $Var\left[D_{1}^{w}\left(p\right)\right]=E\left[{D_{1}^{w}\left(p\right)}^{2}\right]-E{\left[D_{1}^{w}\left(p\right)\right]}^{2}$ :

(6) $$Var\left[D_{1}^{w}\left(p\right)\right]=\sigma_{2}-\sigma_{3}-3\sigma_{2}^{2}+8\sigma_{2}\sigma_{3}-4\sigma_{2}\sigma_{4}-4\sigma_{3}^{2}+4\sigma_{3}\sigma_{4}-\sigma_{4}^{2}.$$

For the *I* = 2 case, we once again use that *p*_2_ = 1 − *p*_1_:

(7) $$E\left[D_{1}^{w}{\left(p\right)}^{2}\right]=p_{1}-p_{1}^{2}$$

(8) $$Var\left[D_{1}^{w}\left(p\right)\right]=p_{1}-5p_{1}^{2}+16p_{1}^{3}-32p_{1}^{4}+40p_{1}^{5}-32p_{1}^{6}+16p_{1}^{7}-4p_{1}^{8}.$$

Figure 1B plots Eq. (8) as a function of *p*_1_. Like the mean, the variance of the dissimilarity increases from 0 at *p*_1_ = 0 to a peak at $p_{1}=\frac{1}{2}$ , decreasing symmetrically to 0 at *p*_1_ = 1. The maximal variance is $\frac{7}{64}$ .

### Distribution of $D_{2}^{w}$

3.2

We compute the distribution of random variable $D_{2}^{w}$ . This computation uses the same probabilities for genotype pairs as those used for $D_{1}^{w}$ in Table 2.

$P\left[D_{2}^{w}=d\right]$ . We compute the probability for each of the possible values of $D_{2}^{w}$ by summing probabilities in Table 2. The resulting probabilities appear in Table 4.

**Table 4:** Probability distribution of $D_{2}^{w}\left(p\right)$ , the allele-sharing dissimilarity $D_{2}^{w}$ for a pair of individuals sampled at random from a population with allele-frequency vector **p**. The table is obtained by summing entries in Table 2.

Value of the dissimilarity (*d*)	$P\left[D_{2}^{w}\left(p\right)=d\right]$
0	*σ*_4_
$\frac{1}{2}$	$4\sigma_{3}+2\sigma_{2}^{2}-6\sigma_{4}$
$\frac{3}{4}$	$4\sigma_{2}-8\sigma_{3}-4{\sigma_{2}}^{2}+8\sigma_{4}$
1	$1-4\sigma_{2}+4\sigma_{3}+2\sigma_{2}^{2}-3\sigma_{4}$

$E\left[D_{2}^{w}\right]$ . Summing across the possible values for the dissimilarity,

$$E\left[D_{2}^{w}\left(p\right)\right]=\sum_{d\in \left\{0,\frac{1}{2},\frac{3}{4},1\right\}}\text{d}P\left[D_{2}^{w}\left(p\right)=d\right],$$

yielding the result

(9) $$E\left[D_{2}^{w}\left(p\right)\right]=1-\sigma_{2}.$$

Note that Eq. (9) gives the “expected heterozygosity,” the probability that two draws from the allele-frequency distribution produce distinct alleles.

For the *I* = 2 case, we have $\sigma_{2}=p_{1}^{2}+{\left(1-p_{1}\right)}^{2}=1-2p_{1}+2p_{1}^{2}$ , so Eq. (9) simplifies to

(10) $$E\left[D_{2}^{w}\left(p\right)\right]=2p_{1}-2p_{1}^{2}=2p_{1}\left(1-p_{1}\right).$$

Figure 1A plots Eq. (10) as a function of *p*_1_. The mean value of the dissimilarity is symmetric around a peak at $\left(\frac{1}{2},\frac{1}{2}\right)$ , equaling 0 at *p*_1_ = 0 and *p*_1_ = 1.

$Var\left[D_{2}^{w}\right]$ . The variance of the distribution of $D_{2}^{w}$ is obtained using $Var\left[D_{2}^{w}\right]=E\left[{D_{2}^{w}\left(p\right)}^{2}\right]-E{\left[D_{2}^{w}\left(p\right)\right]}^{2}$ . We first find

(11) $$\begin{array}{ll} E\left[D_{2}^{w}{\left(p\right)}^{2}\right] & =\sum_{d\in \left\{0,\frac{1}{2},\frac{3}{4},1\right\}}{\text{d}}^{2}P\left[D_{2}^{w}\left(p\right)=d\right]\\ & =1-\frac{7}{4}\sigma_{2}+\frac{1}{2}\sigma_{3}+\frac{1}{4}\sigma_{2}^{2}. \end{array}$$

Therefore,

(12) $$Var\left[D_{2}^{w}\left(p\right)\right]=\frac{1}{4}\sigma_{2}+\frac{1}{2}\sigma_{3}-\frac{3}{4}\sigma_{2}^{2}.$$

For the *I* = 2 case, we use *p*_2_ = 1 − *p*_1_ to obtain

(13) $$E\left[D_{2}^{w}{\left(p\right)}^{2}\right]=p_{1}-2p_{1}^{3}+p_{1}^{4}$$

(14) $$Var\left[D_{2}^{w}\left(p\right)\right]=p_{1}-4p_{1}^{2}+6p_{1}^{3}-3p_{1}^{4}.$$

Figure 1B plots Eq. (14). The variance has peaks at $\left(\frac{3-\sqrt{3}}{6},\frac{1}{12}\right)$ and $\left(\frac{3+\sqrt{3}}{6},\frac{1}{12}\right)$ , between which it has a local minimum at $\left(\frac{1}{2},\frac{1}{16}\right)$ . It equals 0 at *p*_1_ = 0 and *p*_1_ = 1.

### Comparison of $D_{1}^{w}$ and $D_{2}^{w}$

3.3

Comparing $E\left[D_{1}^{w}\right]$ (Eq. (3)) and $E\left[D_{2}^{w}\right]$ (Eq. (9)), we quickly observe that if *p*_*i*_ ≠ 1 for all *i*, then

(15) $$E\left[D_{1}^{w}\right]<E\left[D_{2}^{w}\right].$$

The result follows by noting ${\left(1-p_{i}\right)}^{2}>0$ for all *i*, so that $\sum_{i=1}^{I}p_{i}^{2}\left(2p_{i}\right)<\sum_{i=1}^{I}p_{i}^{2}\left(1+p_{i}^{2}\right)$ and 2*σ*_3_ < *σ*_2_ + *σ*_4_, from which we obtain Eq. (15). In fact, Eq. (15) follows from Table 1: for all possible genotype combinations, $D_{1}^{w}\leq D_{2}^{w}$ , and the inequality is strict in two of seven cases, at least one of which must have nonzero probability if *p*_*i*_ ≠ 1 for all *i*.

**Figure 2:**
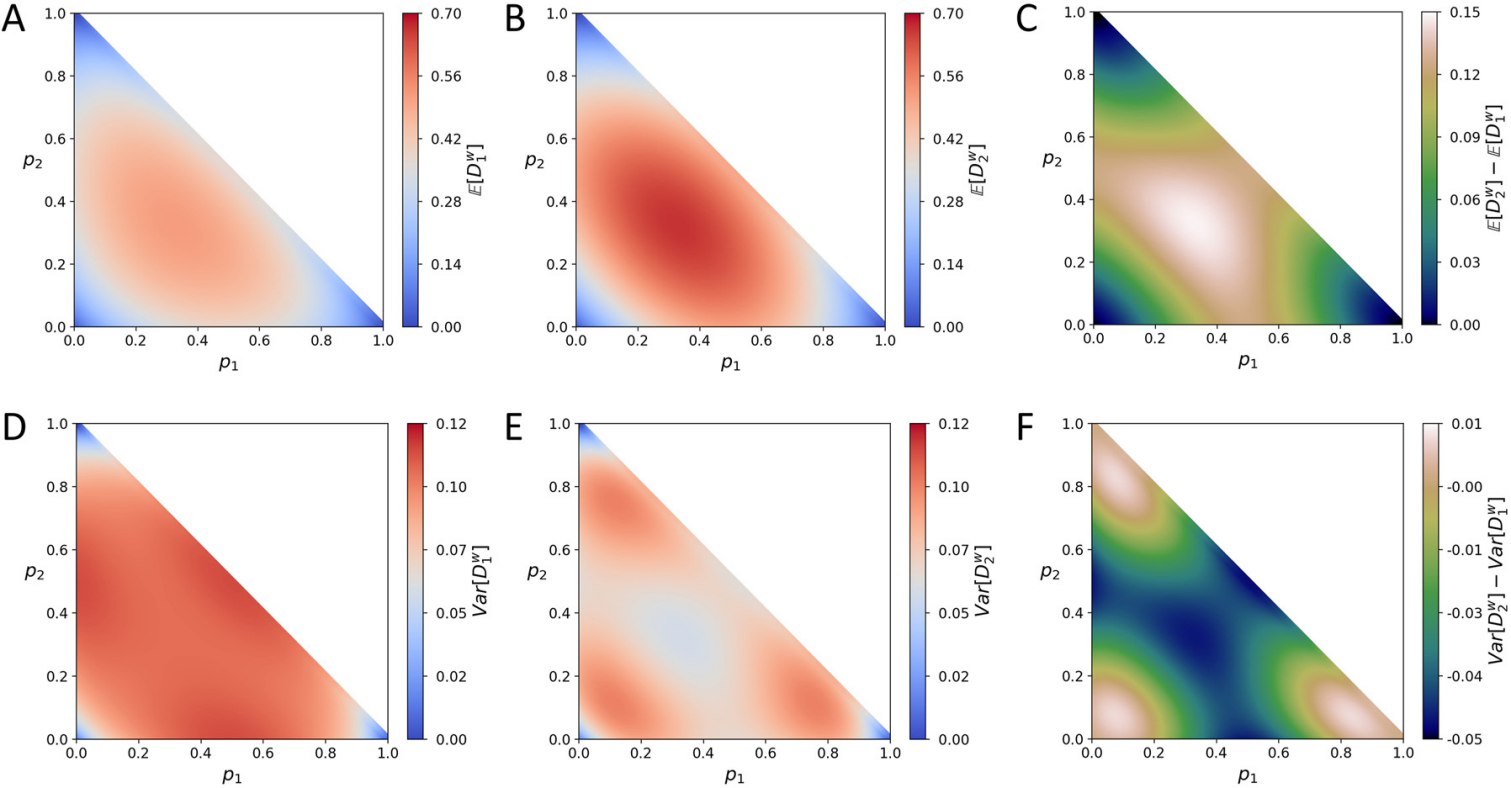
Mean and variance of the within-population dissimilarities $D_{1}^{w}$ and $D_{2}^{w}$ for *I* = 3 alleles as functions of the frequencies *p*_1_ and *p*_2_ of two of the alleles. (A) Mean of $D_{1}^{w}$ , Eq. (3). (B) Mean of $D_{2}^{w}$ , Eq. (9). (C) $E\left[D_{2}^{w}\right]-E\left[D_{1}^{w}\right]$ . (D) Variance of $D_{1}^{w}$ , Eq. (6). (E) Variance of $D_{2}^{w}$ , Eq. (12). (F) $Var\left[D_{2}^{w}\right]-Var\left[D_{1}^{w}\right]$ .

For *I* = 2, Eq. (15) can be observed in Figure 1A, as it can be seen that the curve for $E\left[D_{2}^{w}\right]$ exceeds that for $E\left[D_{1}^{w}\right]$ . The largest excess occurs at $p_{1}=p_{2}=\frac{1}{2}$ . Figure 2C plots the difference $E\left[D_{2}^{w}\right]-E\left[D_{1}^{w}\right]$ for the case of *I* = 3, and the maximal difference in the figure also occurs when alleles have the same frequency, $\left(p_{1},p_{2},p_{3}\right)=\left(\frac{1}{3},\frac{1}{3},\frac{1}{3}\right)$ .

For the variances, Figure 1B finds that for *I* = 2, $Var\left[D_{1}^{w}\right]>Var\left[D_{2}^{w}\right]$ for intermediate *p*_1_, and that the two variances are comparable for *p*_1_ near 0 or 1, with some *p*_1_ values producing $Var\left[D_{1}^{w}\right]<Var\left[D_{2}^{w}\right]$ . Figure 2F illustrates a similar result for *I* = 3. For both *I* = 2 and *I* = 3, at intermediate allele frequencies, $Var\left[D_{1}^{w}\right]>Var\left[D_{2}^{w}\right]$ ; at extreme allele frequencies, the two variances are comparable, sometimes with $Var\left[D_{1}^{w}\right]<Var\left[D_{2}^{w}\right]$ .

## Distribution of $D^{b}$

4

We now examine allele-sharing dissimilarities between pairs of individuals from different populations. Let **p** be the allele frequency vector for the population from which the first individual is sampled, and let **q** be the corresponding vector for the population of the second individual; the special case of **q** = **p** follows Section 3. We evaluate the properties of the random variables $D_{1}^{b}$ and $D_{2}^{b}$ .

### Distribution of $D_{1}^{b}$

4.1

$P\left[D_{1}^{b}=d\right]$ . We obtain the probability for each possible genotype combination for a pair of individuals from different populations. For this computation, we use the polynomials in Eqs. (1) and (2). The resulting probabilities appear in Table 5.

**Table 5:** Probability of genotype combinations for pairs of individuals sampled from two populations. For each case, the probability is written as a sum, which is then simplified using Eqs. (1) and (2).

Case	Genotypes	Probability	Simplified probability
1	*AA*, *AA*	$\overset{I}{\underset{i=1}{\sum }}p_{i}^{2}q_{i}^{2}$	*ρ*_22_
2	*AA*, *AB*	$2\sum_{i=1}^{I}p_{i}^{2}q_{i}\sum_{\begin{array}{r} j=1\\ j\neq i \end{array}}^{I}q_{j}+2\sum_{i=1}^{I}p_{i}q_{i}^{2}\sum_{\begin{array}{r} j=1\\ j\neq i \end{array}}^{I}p_{j}$	2*ρ*_21_ + 2*ρ*_12_ − 4*ρ*_22_
3	*AA*, *BB*	$\sum_{i=1}^{I}p_{i}^{2}\sum_{\begin{array}{r} j=1\\ j\neq i \end{array}}^{I}q_{j}^{2}$	*σ*_2_ *τ*_2_ − *ρ*_22_
4	*AA*, *BC*	$\sum_{i=1}^{I}p_{i}^{2}\sum_{\begin{array}{r} j=1\\ j\neq i \end{array}}^{I}q_{j}\sum_{\begin{array}{r} k=1\\ k\neq i,j \end{array}}^{I}q_{k}+\sum_{i=1}^{I}q_{i}^{2}\sum_{\begin{array}{r} j=1\\ j\neq i \end{array}}^{I}p_{j}\sum_{\begin{array}{r} k=1\\ k\neq i,j \end{array}}^{I}p_{k}$	*σ*_2_ + *τ*_2_ − 2*σ*_2_ *τ*_2_ − 2*ρ*_21_ − 2*ρ*_12_ + 4*ρ*_22_
5	*AB*, *AB*	$2\sum_{i=1}^{I}p_{i}q_{i}\sum_{\begin{array}{r} j=1\\ j\neq i \end{array}}^{I}p_{j}q_{j}$	$2\rho_{11}^{2}-2\rho_{22}$
6	*AB*, *AC*	$4\sum_{i=1}^{I}p_{i}q_{i}\sum_{\begin{array}{r} j=1\\ j\neq i \end{array}}^{I}p_{j}\sum_{\begin{array}{r} k=1\\ k\neq i,j \end{array}}^{I}q_{k}$	$4\rho_{11}-4\rho_{21}-4\rho_{12}-4\rho_{11}^{2}+8\rho_{22}$
7	*AB*, *CD*	$\sum_{i=1}^{I}p_{i}\sum_{\begin{array}{r} j=1\\ j\neq i \end{array}}^{I}p_{j}\sum_{\begin{array}{r} k=1\\ k\neq i,j \end{array}}^{I}q_{k}\sum_{\begin{array}{r} \ell =1\\ \ell \neq i,j,k \end{array}}^{I}q_{\ell }$	$1-\sigma_{2}-\tau_{2}+\sigma_{2}\tau_{2}-4\rho_{11}+4\rho_{21}+4\rho_{12}-6\rho_{22}+2\rho_{11}^{2}$

We sum across genotype combinations to obtain probabilities for $D_{1}^{b}$ to equal particular values. Table 6 provides these probabilities.

**Table 6:** Probability distribution of $D_{1}^{b}\left(p,q\right)$ , the allele-sharing dissimilarity $D_{1}^{b}$ for a pair of individuals sampled at random from two populations with allele-frequency vectors **p** and **q**. The table is obtained by summing entries in Table 5.

Value of the dissimilarity (*d*)	$P\left[D_{1}^{b}\left(p,q\right)=d\right]$
0	$2\rho_{11}^{2}-\rho_{22}$
$\frac{1}{2}$	$4\rho_{11}-2\rho_{21}-2\rho_{12}-4\rho_{11}^{2}+4\rho_{22}$
1	$1-4\rho_{11}+2\rho_{21}+2\rho_{12}+2\rho_{11}^{2}-3\rho_{22}$

$E\left[D_{1}^{b}\right]$ . As we did for the within-population dissimilarity $D_{1}^{w}\left(p\right)$ , we compute the expected value of the distribution of the between-population dissimilarity $D_{1}^{b}\left(p,q\right)$ as

$$E\left[D_{1}^{b}\left(p,q\right)\right]=\sum_{d\in \left\{0,\frac{1}{2},1\right\}}dP\left[D_{1}^{b}\left(p,q\right)=d\right].$$

Using the values in Table 6, we obtain

(16) $$E\left[D_{1}^{b}\left(p,q\right)\right]=1-2\rho_{11}+\rho_{21}+\rho_{12}-\rho_{22}.$$

For the *I* = 2 case, with *p*_2_ = 1 − *p*_1_ and *q*_2_ = 1 − *q*_1_, Eq. (16) simplifies to

(17) $$E\left[D_{1}^{b}\left(p,q\right)\right]=p_{1}+q_{1}-4p_{1}q_{1}+2p_{1}^{2}q_{1}+2p_{1}q_{1}^{2}-2p_{1}^{2}q_{1}^{2}.$$

**Figure 3:**
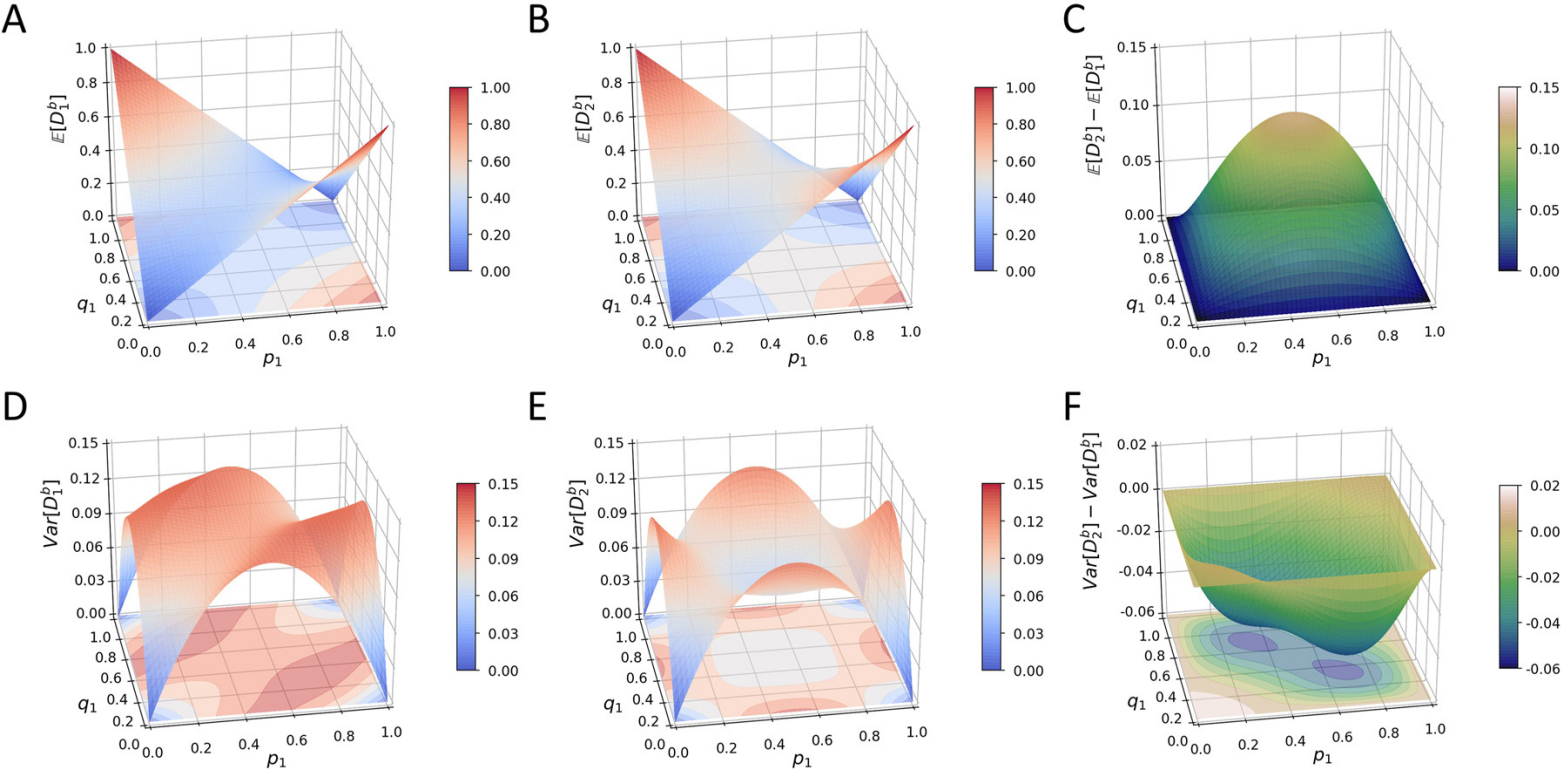
Mean and variance of the between-population dissimilarities $D_{1}^{b}$ and $D_{2}^{b}$ for *I* = 2 alleles as functions of the frequencies (*p*_1_, *q*_1_) of one of the alleles. (A) Mean of $D_{1}^{b}$ , Eq. (17). (B) Mean of $D_{2}^{b}$ , Eq. (23). (C) $E\left[D_{2}^{b}\right]-E\left[D_{1}^{b}\right]$ . (D) Variance of $D_{1}^{b}$ , Eq. (21). (E) Variance of $D_{2}^{b}$ , Eq. (27). (F) $Var\left[D_{2}^{b}\right]-Var\left[D_{1}^{b}\right]$ .

Figure 3A plots Eq. (17). The figure has maxima of 1 at (*p*_1_, *q*_1_) = (1, 0) and (0,1), when the two populations have the greatest difference in allele frequency, and equals 0 at (0,0) and (1,1). It has a saddle surface with a value of $\frac{3}{8}$ at saddle point $\left(p_{1},q_{1}\right)=\left(\frac{1}{2},\frac{1}{2}\right)$ .

$Var\left[D_{1}^{b}\right]$ . We first compute

(18) $$\begin{array}{ll} E\left[D_{1}^{b}{\left(p,q\right)}^{2}\right] & =\sum_{d\in \left\{0,\frac{1}{2},1\right\}}{\text{d}}^{2}P\left[D_{1}^{b}\left(p,q\right)=d\right]\\ & =1-3\rho_{11}+\frac{3}{2}\rho_{21}+\frac{3}{2}\rho_{12}-2\rho_{22}+\rho_{11}^{2}. \end{array}$$

Using $Var\left[D_{1}^{b}\left(p,q\right)\right]=E\left[{D_{1}^{b}\left(p,q\right)}^{2}\right]-E{\left[D_{1}^{b}\left(p,q\right)\right]}^{2}$ , the variance is thus

(19) $$\begin{array}{ll} Var\left[D_{1}^{b}\left(p,q\right)\right] & =\rho_{11}-\frac{1}{2}\rho_{21}-\frac{1}{2}\rho_{12}-3\rho_{11}^{2}+4\rho_{11}\rho_{21}+4\rho_{11}\rho_{12}-4\rho_{11}\rho_{22}-\rho_{21}^{2}-\rho_{12}^{2}-2\rho_{12}\rho_{21}\\ & \;+2\rho_{12}\rho_{22}+2\rho_{21}\rho_{22}-\rho_{22}^{2}. \end{array}$$

For the *I* = 2 case, we have *p*_1_ = 1 − *p*_2_ and *q*_1_ = 1 − *q*_2_. Equations (18) and (19) simplify to

(20) $$E\left[D_{1}^{b}{\left(p,q\right)}^{2}\right]=\frac{1}{2}p_{1}+\frac{1}{2}q_{1}-2p_{1}q_{1}+\frac{1}{2}p_{1}^{2}+\frac{1}{2}q_{1}^{2}$$

(21) $$\begin{array}{ll} Var\left[D_{1}^{b}\left(p,q\right)\right] & =\frac{1}{2}p_{1}+\frac{1}{2}q_{1}-4p_{1}q_{1}-\frac{1}{2}p_{1}^{2}-\frac{1}{2}q_{1}^{2}+8p_{1}^{2}q_{1}+8p_{1}q_{1}^{2}-4p_{1}^{3}q_{1}-24p_{1}^{2}q_{1}^{2}-4p_{1}q_{1}^{3}\\ & \;+20p_{1}^{3}q_{1}^{2}+20p_{1}^{2}q_{1}^{3}-4p_{1}^{4}q_{1}^{2}-24p_{1}^{3}q_{1}^{3}-4p_{1}^{2}q_{1}^{4}+8p_{1}^{4}q_{1}^{3}+8p_{1}^{3}q_{1}^{4}-4p_{1}^{4}q_{1}^{4}. \end{array}$$

Figure 3D shows that the variance has higher values away from the four corners (0,0), (1,0), (0,1), and (1,1) for (*p*_1_, *q*_1_), equaling 0 in each of these corners.

### Distribution of $D_{2}^{b}$

4.2

$P\left[D_{2}^{b}=d\right]$ . We use Table 5 to obtain the probabilities of particular values of $D_{2}^{b}$ . The resulting probabilities appear in Table 7.

**Table 7:** Probability distribution of $D_{2}^{b}\left(p,q\right)$ , the allele-sharing dissimilarity $D_{2}^{b}$ for a pair of individuals sampled at random from two populations with allele-frequency vectors **p** and **q**. The table is obtained by summing entries in Table 5.

Value of the dissimilarity (*d*)	$P\left[D_{2}^{b}\left(p,q\right)=d\right]$
0	*ρ*_22_
$\frac{1}{2}$	$2\rho_{21}+2\rho_{12}+2\rho_{11}^{2}-6\rho_{22}$
$\frac{3}{4}$	$4\rho_{11}-4\rho_{21}-4\rho_{12}-4\rho_{11}^{2}+8\rho_{22}$
1	$1-4\rho_{11}+2\rho_{21}+2\rho_{12}+2\rho_{11}^{2}-3\rho_{22}$

$E\left[D_{2}^{b}\right]$ . For $D_{2}^{b}$ , we substitute the values from Table 7 into

$$E\left[D_{2}^{b}\left(p,q\right)\right]=\sum_{d\in \left\{0,\frac{1}{2},\frac{3}{4},1\right\}}dP\left[D_{2}^{b}\left(p,q\right)=d\right].$$

We obtain

(22) $$E\left[D_{2}^{b}\left(p,q\right)\right]=1-\rho_{11}.$$

This quantity is the between-population analogue of expected heterozygosity, the probability that two random draws, one from the allele-frequency distribution of a locus in one population and one from the corresponding distribution in a second population, represent the same allele.

For the *I* = 2 case, Eq. (22) simplifies to

(23) $$E\left[D_{2}^{b}\left(p,q\right)\right]=p_{1}+q_{1}-2p_{1}q_{1}.$$

Figure 3B plots Eq. (23). The figure has maxima of 1 at (*p*_1_, *q*_1_) = (1, 0) and (0,1) and equals 0 at (0,0) and (1,1). It has a saddle surface with a value of $\frac{1}{2}$ at saddle point $\left(p_{1},q_{1}\right)=\left(\frac{1}{2},\frac{1}{2}\right)$ .

$Var\left[D_{2}^{b}\right]$ . We find that

(24) $$\begin{array}{ll} E\left[D_{2}^{b}{\left(p,q\right)}^{2}\right] & =\sum_{d\in \left\{0,\frac{1}{2},1\right\}}{\text{d}}^{2}P\left[D_{2}^{b}\left(p,q\right)=d\right]\\ & =1-\frac{7}{4}\rho_{11}+\frac{1}{4}\rho_{21}+\frac{1}{4}\rho_{12}+\frac{1}{4}\rho_{11}^{2}. \end{array}$$

Therefore, by $Var\left[D_{2}^{b}\left(p,q\right)\right]=E\left[{D_{2}^{b}\left(p,q\right)}^{2}\right]-E{\left[D_{2}^{b}\left(p,q\right)\right]}^{2}$ ,

(25) $$Var\left[D_{2}^{b}\left(p,q\right)\right]=\frac{1}{4}\rho_{11}+\frac{1}{4}\rho_{21}+\frac{1}{4}\rho_{12}-\frac{3}{4}\rho_{11}^{2}.$$

For the *I* = 2 case, Eqs. (24) and (25) simplify to

(26) $$E\left[D_{2}^{b}{\left(p,q\right)}^{2}\right]=\frac{1}{2}p_{1}+\frac{1}{2}q_{1}+\frac{1}{2}p_{1}^{2}+\frac{1}{2}q_{1}^{2}-p_{1}q_{1}-p_{1}^{2}q_{1}-p_{1}q_{1}^{2}+p_{1}^{2}q_{1}^{2}$$

(27) $$Var\left[D_{2}^{b}\left(p,q\right)\right]=\frac{1}{2}p_{1}+\frac{1}{2}q_{1}-3p_{1}q_{1}-\frac{1}{2}p_{1}^{2}-\frac{1}{2}q_{1}^{2}+3p_{1}^{2}q_{1}+3p_{1}q_{1}^{2}-3p_{1}^{2}q_{1}^{2}.$$

Figure 3E plots Eq. (27). The variance is greatest at $\left(p_{1},q_{1}\right)=\left(\frac{1}{2},0\right)$ , $\left(\frac{1}{2},1\right)$ , $\left(0,\frac{1}{2}\right)$ , and $\left(1,\frac{1}{2}\right)$ and equals 0 at (0,0), (1,0), (0,1), and (1,1). It has a local minimum at $\left(p_{1},q_{1}\right)=\left(\frac{1}{2},\frac{1}{2}\right)$ .

### Comparison of $D_{1}^{b}$ and $D_{2}^{b}$

4.3

The two measures for the between-population dissimilarity have the same expected value, $E\left[D_{1}^{b}\right]=E\left[D_{2}^{b}\right]$ , if for all *i*, at least one of *p*_*i*_, 1 − *p*_*i*_, *q*_*i*_, and 1 − *q*_*i*_ is zero. The condition for equality can be seen from $E\left[D_{2}^{b}\right]-E\left[D_{1}^{b}\right]=\rho_{11}-\rho_{21}-\rho_{12}+\rho_{22}=\sum_{i=1}^{I}p_{i}\left(1-p_{i}\right)q_{i}\left(1-q_{i}\right)$ . Excluding these equality cases, we have

(28) $$E\left[D_{1}^{b}\right]<E\left[D_{2}^{b}\right].$$

Note that $D_{1}^{b}\leq D_{2}^{b}$ for all possible genotype combinations in Table 1.

The inequality in Eq. (28) can be observed for the *I* = 2 case in Figure 3C, where the surface plot of $E\left[D_{2}^{b}\right]-E\left[D_{1}^{b}\right]$ remains greater than or equal to 0, with equality only on the boundary. The largest difference occurs at $p_{1}=q_{1}=\frac{1}{2}$ .

Figure 3F compares the variances of $D_{1}^{b}$ and $D_{2}^{b}$ for the case of *I* = 2. Across most of the parameter space, $Var\left[D_{1}^{b}\right]>Var\left[D_{2}^{b}\right]$ . The excess is greatest at points $\left(p_{1},q_{1}\right)=\left(\frac{1}{3},\frac{2}{3}\right)$ and $\left(\frac{2}{3},\frac{1}{3}\right)$ .

## The relative magnitudes of $E\left[D^{w}\right]$ and $E\left[D^{b}\right]$

5

We now examine the relative magnitudes of the expectations $E\left[D^{w}\right]$ and $E\left[D^{b}\right]$ . We determine the conditions under which the expectation of a within-population dissimilarity exceeds that of a between-population dissimilarity.

### Inequality relationship between $E\left[D_{1}^{w}\left(p\right)\right]$ and $E\left[D_{1}^{b}\left(p,q\right)\right]$

5.1

For arbitrary *I*, using Eqs. (3) and (16), the expression $E\left[D_{1}^{w}\left(p\right)\right]>E\left[D_{1}^{b}\left(p,q\right)\right]$ is equivalent to

(29) $$-2\sigma_{2}+2\sigma_{3}-\sigma_{4}+2\rho_{11}-\rho_{21}-\rho_{12}+\rho_{22}>0.$$

This condition can be written with vector notation. Let $\tilde{p}=\left(p_{1}^{2},p_{2}^{2},\ldots ,p_{I}^{2}\right)$ and $\tilde{q}=\left(q_{1}^{2},q_{2}^{2},\ldots ,q_{I}^{2}\right)$ , treating **p**, **q**, $\tilde{p}$ , and $\tilde{q}$ as row vectors. We have the identities *σ*_2_ = **pp** ^*T*^, $\sigma_{3}=p{\tilde{p}}^{T}=\tilde{p}p^{T}$ , $\sigma_{4}=\tilde{p}{\tilde{p}}^{T}$ , *ρ*_11_ = **pq** ^*T*^, $\rho_{12}=p{\tilde{q}}^{T}$ , $\rho_{21}=\tilde{p}q^{T}$ , and $\rho_{22}=\tilde{p}{\tilde{q}}^{T}$ .

Equation (29) thus becomes

(30) $$-2pp^{T}+2p{\tilde{p}}^{T}-\tilde{p}{\tilde{p}}^{T}+2pq^{T}-\tilde{p}q^{T}-p{\tilde{q}}^{T}+\tilde{p}{\tilde{q}}^{T}>0,$$

which simplifies to

(31) $$\left(\begin{array}{cc} p & p-\tilde{p} \end{array}\right)\left(\begin{array}{c} {\left(p-q\right)}^{T}\\ {\left[\left(p-\tilde{p}\right)-\left(q-\tilde{q}\right)\right]}^{T} \end{array}\right)<0.$$

For *I* = 2, we can further simplify this condition on *p*_1_ and *q*_1_, noting *p*_2_ = 1 − *p*_1_ and *q*_2_ = 1 − *q*_1_.

**Theorem 1**

Consider a locus with *I* = 2 distinct alleles. For individuals sampled from two populations with allele frequency vectors **p** = (*p*_1_, 1 − *p*_1_) and **q** = (*q*_1_, 1 − *q*_1_), $E\left[D_{1}^{w}\left(p\right)\right]>E\left[D_{1}^{b}\left(p,q\right)\right]$ holds if and only if

(32) $$\left\{\begin{array}{cc} 0<q_{1}<p_{1}\; & \;\text{ if }0<p_{1}\leq a,\\ g\left(p_{1}\right)<q_{1}<p_{1}\; & \;\text{ if }a\leq p_{1}<\frac{1}{2},\\ p_{1}<q_{1}<g\left(p_{1}\right)\; & \;\text{ if }\frac{1}{2}<p_{1}\leq 1-a,\\ p_{1}<q_{1}<1\; & \;\text{ if }1-a\leq p_{1}<1, \end{array}\right.$$

where

$$g\left(x\right)=\frac{2x^{3}-4x^{2}+4x-1}{2x\left(1-x\right)},$$

and

$$a=\frac{1}{3}\left(\frac{\sqrt[{3}]{3\sqrt{33}-13}}{2^{2/3}}-\frac{2^{5/3}}{\sqrt[{3}]{3\sqrt{33}-13}}+2\right)\approx 0.3522$$

is the unique real root of 2*x* ^3^ − 4*x* ^2^ + 4*x* − 1.

**Proof**

We simplify Eq. (29) noting *p*_2_ = 1 − *p*_1_ and *q*_2_ = 1 − *q*_1_. To find the region where $E\left[D_{1}^{w}\left(p\right)\right]>E\left[D_{1}^{b}\left(p,q\right)\right]$ , we solve the polynomial inequality

(33) $$p_{1}-q_{1}-4p_{1}^{2}+4p_{1}q_{1}+4p_{1}^{3}-2p_{1}^{2}q_{1}-2p_{1}q_{1}^{2}-2p_{1}^{4}+2p_{1}^{2}q_{1}^{2}>0,$$

with 0 ≤ *p*_1_ ≤ 1 and 0 ≤ *q*_1_ ≤ 1. Solving for *q*_1_ in terms of *p*_1_, we find that the expression in Eq. (33) is 0 at *q*_1_ = *p*_1_ and at *q*_1_ = *g*(*p*_1_), and for fixed *p*, it is positive when *q* lies between the two roots. The unique real root for *g*(*x*) = *x* is at $x=\frac{1}{2}$ , so that *g*(*p*_1_) < *p*_1_ for $p_{1}<\frac{1}{2}$ and *g*(*p*_1_) > *p*_1_ for $p_{1}>\frac{1}{2}$ .

For $0\leq p_{1}<\frac{1}{2}$ , *g*(*p*_1_) < 0 for *p*_1_ < *a*, so that for 0 ≤ *p*_1_ ≤ *a*, the region where the expression in Eq. (33) is positive includes the full interval (0, *p*_1_) for *q*_1_. For $a\leq p_{1}\leq \frac{1}{2}$ , it is positive only in interval (*g*(*p*_1_), *p*_1_) for *q*_1_.

For $\frac{1}{2}<p_{1}<1$ , *g*(*p*_1_) = 1 for *p*_1_ = 1 − *a*, with *g*(*p*_1_) < 1 for *p*_1_ in $\left[\frac{1}{2},1-a\right)$ and *g*(*p*_1_) > 1 for *p*_1_ in $\left(1-a,1\right]$ . Hence, for *p*_1_ in $\left[\frac{1}{2},1-a\right]$ , the expression in Eq. (33) is positive for *q*_1_ in (*p*_1_, *g*(*p*_1_)), and for *p*_1_ in [1 − *a*, 1], it is positive for *q*_1_ in (*p*_1_, 1). □

**Figure 4:**
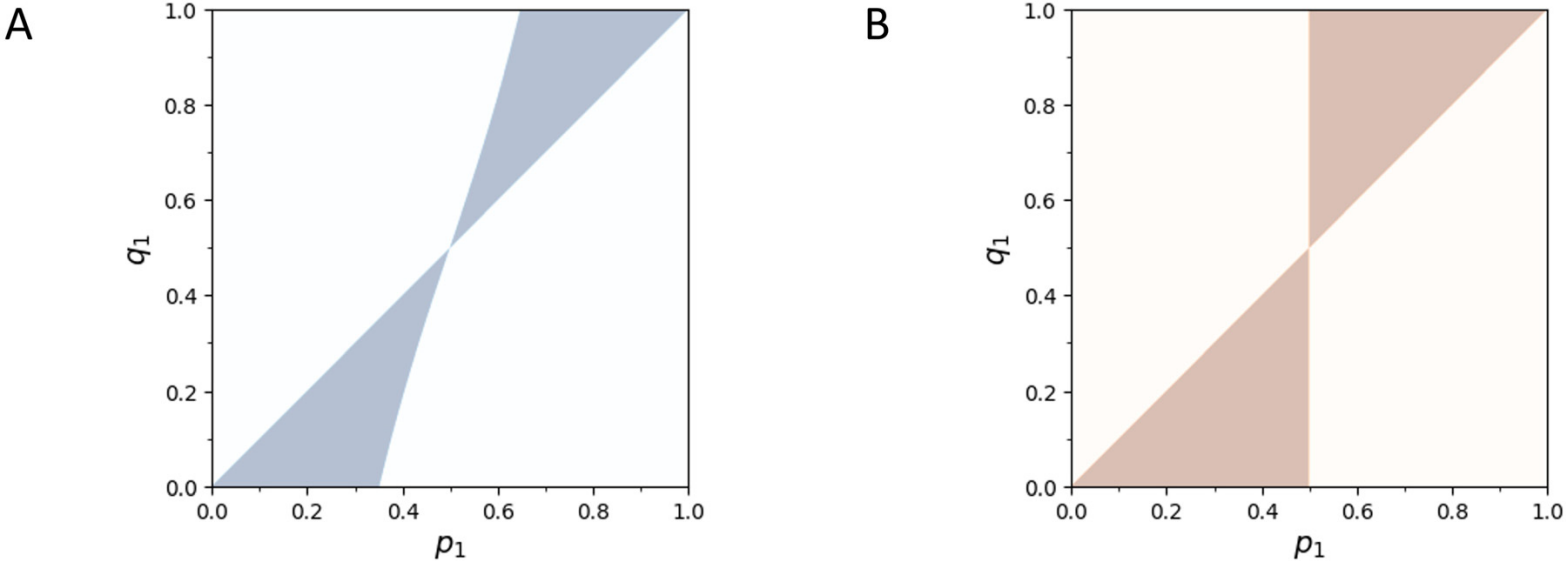
Values of (*p*_1_, *q*_1_) for which $E\left[D^{w}\right]>E\left[D^{b}\right]$ in the case of *I* = 2 alleles, shaded in color. (A) $D_{1}$ , Theorem 1. (B) $D_{2}$ , Theorem 2.

Figure 4A plots the region identified in Theorem 1. That a nonempty region exists indicates that sometimes, allele frequencies for a biallelic locus produce a within-population dissimilarity that exceeds the between-population dissimilarity. Note that because the choice of which allele is labeled 1 and which is labeled 2 is arbitrary, (*p*_1_, *q*_1_) is included in the region if and only if (1 − *p*_1_, 1 − *q*_1_) is also included.

We can calculate the area of the region in the unit square representing the probability $P\left(E\left[D_{1}^{w}\right]>E\left[D_{1}^{b}\right]\right)$ under the assumption that *p*_1_ and *q*_1_ are independently and identically distributed with uniform-[0,1] distribution:

(34) $$\begin{array}{ll} & P\left(E\left[D_{1}^{w}\right]>E\left[D_{1}^{b}\right]\right)\\ & \;=\overset{a}{\underset{p_{1}=0}{\int }}\overset{p_{1}}{\underset{q_{1}=0}{\int }}1\;\text{d}q_{1}\;\text{d}p_{1}+\overset{\frac{1}{2}}{\underset{p_{1}=a}{\int }}\overset{p_{1}}{\underset{q_{1}=g\left(p_{1}\right)}{\int }}1\;\text{d}q_{1}\;\text{d}p_{1}+\overset{1-a}{\underset{p_{1}=\frac{1}{2}}{\int }}\overset{g\left(p_{1}\right)}{\underset{q_{1}=p_{1}}{\int }}1\;\text{d}q_{1}\;\text{d}p_{1}+\overset{1}{\underset{p_{1}=1-a}{\int }}\overset{1}{\underset{q_{1}=p_{1}}{\int }}1\;\text{d}q_{1}\;\text{d}p_{1}\\ & \;=2\left[\overset{a}{\underset{p_{1}=0}{\int }}p_{1}\;\text{d}p_{1}+\overset{\frac{1}{2}}{\underset{p_{1}=a}{\int }}\frac{-4p_{1}^{3}+6p_{1}^{2}-4p_{1}+1}{2p_{1}\left(1-p_{1}\right)}\;\text{d}p_{1}\right]\\ & \;=-a^{2}+2a-\frac{1}{2}-2\log2-\log a-\log\left(1-a\right)\\ & \;\approx 0.17179. \end{array}$$

To evaluate $P\left(E\left[D_{1}^{w}\right]>E\left[D_{1}^{b}\right]\right)$ more generally, for each *I* from 2 to 20, we perform a simulation. In particular, for each *I*, we consider independently and identically distributed vectors **p** and **q** from the uniform distribution over the simplex Δ^*I*−1^ (the Dirichlet-(1, 1, …, 1) distribution, where the vector of 1’s has length *I*). We sample 100,000 replicate pairs (**p**, **q**), and for each pair we evaluate if $E\left[D_{1}^{w}\right]>E\left[D_{1}^{b}\right]$ .

**Figure 5:**
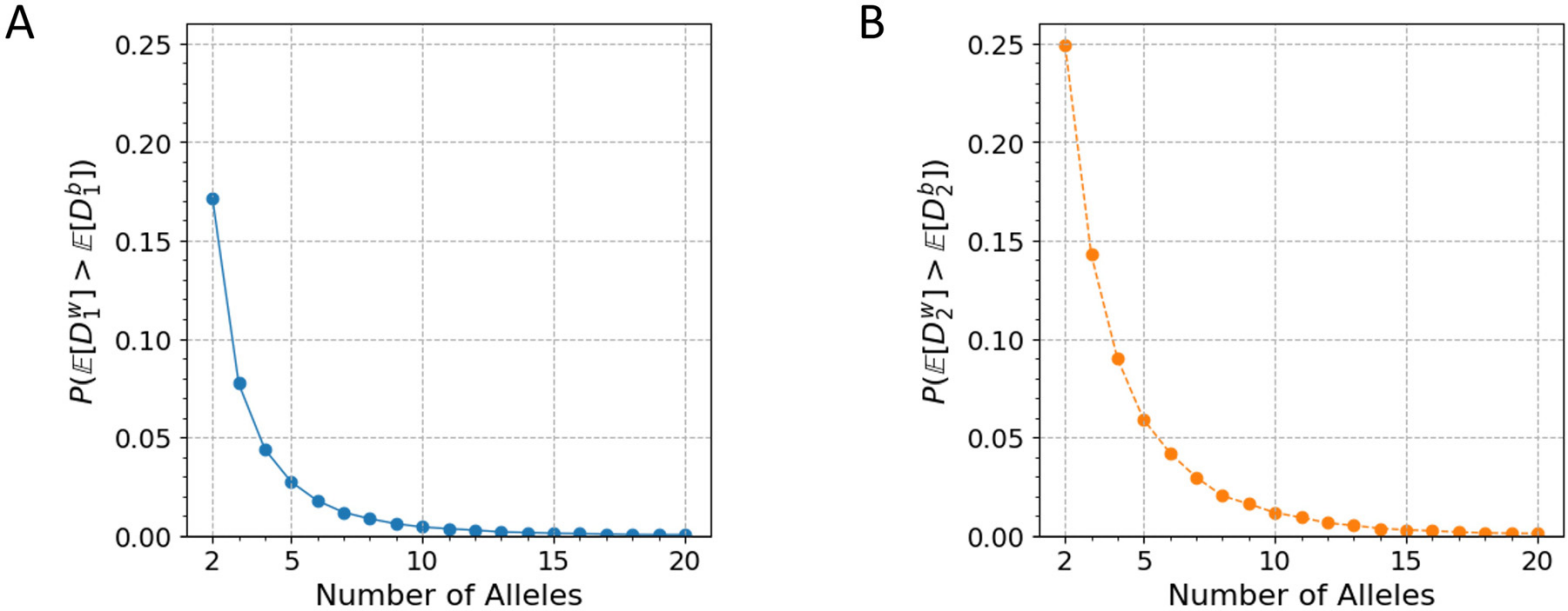
The probability $P\left(E\left[D^{w}\right]>E\left[D^{b}\right]\right)$ for simulated pairs of allele frequency vectors (**p**, **q**) with *I* distinct alleles. (A) $D_{1}$ . (B) $D_{2}$ . Independent and identical uniform distributions are simulated for each *I*, 2 ≤ *I* ≤ 20, by drawing uniformly from the simplex Δ^*I*−1^ (100,000 replicates).

Figure 5A plots the resulting probability. We can observe that for *I* = 2, the simulated $P\left(E\left[D_{1}^{w}\right]>E\left[D_{1}^{b}\right]\right)$ accords with the analytical value in Eq. (34). The probability then decreases with increasing *I*.

### Inequality relationship between $E\left[D_{2}^{w}\left(p\right)\right]$ and $E\left[D_{2}^{b}\left(p,q\right)\right]$

5.2

For arbitrary *I*, via Eqs. (9) and (22), the expression $E\left[D_{2}^{w}\left(p\right)\right]>E\left[D_{2}^{b}\left(p,q\right)\right]$ is equivalent to

(35) $$\rho_{11}-\sigma_{2}>0.$$

With *σ*_2_ = **pp** ^*T*^ and *ρ*_11_ = **pq** ^*T*^, Eq. (35) thus becomes

(36) $$p{\left(p-q\right)}^{T}<0.$$

For *I* = 2, Eq. (36) can be simplified to a condition on *p*_1_ and *q*_1_, again noting *p*_2_ = 1 − *p*_1_ and *q*_2_ = 1 − *q*_1_.

**Theorem 2**

Consider a locus with *I* = 2 distinct alleles. For individuals sampled from two populations with allele frequency vectors **p** = (*p*_1_, 1 − *p*_1_) and **q** = (*q*_1_, 1 − *q*_1_), $E\left[D_{2}^{w}\left(p\right)\right]>E\left[D_{2}^{b}\left(p,q\right)\right]$ holds if and only if

(37) $$\left\{\begin{array}{cc} 0<q_{1}<p_{1}\; & \;\text{ if }0<p_{1}<\frac{1}{2},\\ p_{1}<q_{1}<1\; & \;\text{ if }\frac{1}{2}<p_{1}<1. \end{array}\right.$$

**Proof**

With *p*_2_ = 1 − *p*_1_ and *q*_2_ = 1 − *q*_1_, Eq. (35) simplifies to

$$p_{1}-q_{1}-2p_{1}^{2}+2p_{1}q_{1}>0.$$

Solving this inequality, we arrive at the result. □

Figure 4B plots the region identified in Theorem 2. This region describes the locations in which allele frequencies for a biallelic locus produce a within-population dissimilarity that exceeds the between-population dissimilarity. As is true for $D_{1}$ , (*p*_1_, *q*_1_) is included in the region if and only if (1 − *p*_1_, 1 − *q*_1_) is also included.

The area of the region in the unit square, representing $P\left(E\left[D_{2}^{w}\right]>E\left[D_{2}^{b}\right]\right)$ under the assumption that *p*_1_ and *q*_1_ are independently and identically distributed with uniform-[0,1] distribution, is straightforward:

(38) $$\begin{array}{ll} & P\left(E\left[D_{2}^{w}\right]>E\left[D_{2}^{b}\right]\right)\\ & \;=\overset{\frac{1}{2}}{\underset{p_{1}=0}{\int }}\overset{p_{1}}{\underset{q_{1}=0}{\int }}1\;\text{d}q_{1}\;\text{d}p_{1}+\overset{1}{\underset{p_{1}=\frac{1}{2}}{\int }}\overset{1}{\underset{q_{1}=p_{1}}{\int }}1\;\text{d}q_{1}\;\text{d}p_{1}\\ & \;=\frac{1}{4}. \end{array}$$

We evaluate $P\left(E\left[D_{2}^{w}\right]>E\left[D_{2}^{b}\right]\right)$ for each *I* from 2 to 20 by simulation. For each *I*, we consider independently and identically distributed vectors **p** and **q** from the uniform distribution over the simplex Δ^*I*−1^ (the Dirichlet-(1, 1, …, 1) distribution), sampling 100,000 replicate pairs (**p**, **q**), and evaluating the fraction of pairs for which $E\left[D_{2}^{w}\right]>E\left[D_{2}^{b}\right]$ .

Figure 5B plots the resulting probability, illustrating the agreement between the simulated $P\left(E\left[D_{2}^{w}\right]>E\left[D_{2}^{b}\right]\right)$ and the analytical value in Eq. (38) for *I* = 2. The probability then decreases as *I* increases.

### Comparison of the $E\left[D^{w}\right]-E\left[D^{b}\right]$ inequalities for $D_{1}$ and $D_{2}$

5.3

The inequality $E\left[D^{w}\right]>E\left[D^{b}\right]$ , where the mean dissimilarity between individuals from the same population exceeds that between individuals from different populations, holds under different scenarios for $D_{1}$ and $D_{2}$ . Comparing Eqs. (34) and (38), we see that for the case of *I* = 2, $E\left[D_{1}^{w}\right]>E\left[D_{1}^{b}\right]$ holds over a smaller fraction of the parameter space than the corresponding inequality $E\left[D_{2}^{w}\right]>E\left[D_{2}^{b}\right]$ (Figure 4). Further, if the former inequality holds, then the latter always holds as well.

In Figure 5, we also observe that the probabilities $P\left(E\left[D^{w}\right]>E\left[D^{b}\right]\right)$ are higher for $D_{2}$ than for $D_{1}$ in simulations with different numbers of alleles. Hence, use of $D_{2}$ rather than $D_{1}$ produces a greater probability that the within-population genetic dissimilarity exceeds the between-population dissimilarity.

## The relative magnitudes of $\bar{E\left[D^{w}\right]}$ and $E\left[D^{b}\right]$

6

We have seen that both for $D_{1}$ and for $D_{2}$ , it is possible for the expected dissimilarity $E\left[D^{w}\right]$ of random pairs of individuals within a population to exceed the expected dissimilarity $E\left[D^{b}\right]$ of random pairs between that population and a second population. However, we will see that for a pair of populations, the mean of their two within-population dissimilarities never exceeds their between-population dissimilarity.

For a pair of populations with allele frequency vectors **p** and **q**, let $\bar{E\left[D_{1}^{w}\left(p,q\right)\right]}=\frac{1}{2}\left(E\left[D_{1}^{w}\left(p\right)\right]+E\left[D_{1}^{w}\left(q\right)\right]\right)$ , and let $\bar{E\left[D_{2}^{w}\left(p,q\right)\right]}=\frac{1}{2}\left(E\left[D_{2}^{w}\left(p\right)\right]+E\left[D_{2}^{w}\left(q\right)\right]\right)$ .

### Inequality relationship between $\bar{E\left[D_{1}^{w}\right]\left(p,q\right)}$ and $E\left[D_{1}^{b}\left(p,q\right)\right]$

6.1

**Theorem 3**

$\bar{E\left[D_{1}^{w}\left(p,q\right)\right]}\leq E\left[D_{1}^{b}\left(p,q\right)\right]$ , with equality if and only if **p** = **q**.

**Proof**

We use Eqs. (3) and (16) to rewrite $\bar{E\left[D_{1}^{w}\left(p,q\right)\right]}-E\left[D_{1}^{b}\left(p,q\right)\right]$ , obtaining

$$\begin{array}{ll} & \frac{E\left[D_{1}^{w}\left(p\right)\right]+E\left[D_{1}^{w}\left(q\right)\right]}{2}-E\left[D_{1}^{b}\left(p,q\right)\right]\\ & \;=-\sigma_{2}+\sigma_{3}-\frac{1}{2}\sigma_{4}-\tau_{2}+\tau_{3}-\frac{1}{2}\tau_{4}-\rho_{21}-\rho_{12}+\rho_{22}+2\rho_{11}. \end{array}$$

Rewriting in terms of the vectors **p**, **q**, $\tilde{p}$ , and $\tilde{q}$ , we have

$$\begin{array}{ll} & \frac{E\left[D_{1}^{w}\left(p\right)\right]+E\left[D_{1}^{w}\left(q\right)\right]}{2}-E\left[D_{1}^{b}\left(p,q\right)\right]\\ & \;=-\left(p-q\right){\left(p-q\right)}^{T}+\left(p-q\right){\left(\tilde{p}-\tilde{q}\right)}^{T}-\frac{1}{2}\left(\tilde{p}-\tilde{q}\right){\left(\tilde{p}-\tilde{q}\right)}^{T}\\ & \;=-\frac{1}{2}\|p-q{\|}^{2}-\frac{1}{2}\|\left(p-q\right)-\left(\tilde{p}-\tilde{q}\right){\|}^{2}\\ & \;\leq 0. \end{array}$$

Equality is reached in the last step if and only if **p** = **q**. □

### Inequality relationship between $\bar{E\left[D_{2}^{w}\left(p,q\right)\right]}$ and $E\left[D_{2}^{b}\left(p,q\right)\right]$

6.2

**Theorem 4**

$\bar{E\left[D_{2}^{w}\left(p,q\right)\right]}\leq E\left[D_{2}^{b}\left(p,q\right)\right]$ , with equality if and only if **p** = **q**.

**Proof**

We rewrite $\bar{E\left[D_{2}^{w}\left(p,q\right)\right]}-E\left[D_{2}^{b}\left(p,q\right)\right]$ using Eqs. (9) and (22):

$$\begin{array}{ll} & \frac{E\left[D_{2}^{w}\left(p\right)\right]+E\left[D_{2}^{w}\left(q\right)\right]}{2}-E\left[D_{2}^{b}\left(p,q\right)\right]\\ & \;=-\frac{\sigma_{2}}{2}-\frac{\tau_{2}}{2}+\rho_{11}. \end{array}$$

In terms of the vectors **p** and **q**, we have

$$\begin{array}{ll} & \frac{E\left[D_{2}^{w}\left(p\right)\right]+E\left[D_{2}^{w}\left(q\right)\right]}{2}-E\left[D_{2}^{b}\left(p,q\right)\right]\\ & \;=-\frac{1}{2}pp^{T}-\frac{1}{2}qq^{T}+pq^{T}\\ & \;=-\frac{1}{2}\|p-q{\|}^{2}\\ & \;\leq 0, \end{array}$$

with equality if and only if **p** = **q**. □

### Comparison of the $\bar{E\left[D^{w}\right]}-E\left[D^{b}\right]$ inequalities for $D_{1}$ and $D_{2}$

6.3

The inequality $\bar{E\left[D^{w}\left(p,q\right)\right]}\leq E\left[D^{b}\left(p,q\right)\right]$ , with equality if and only if **p** = **q**, holds for both $D_{1}$ and $D_{2}$ . Comparing the proofs of Theorems 3 and 4, we see that

(39) $$\bar{E\left[D_{1}^{w}\left(p,q\right)\right]}-E\left[D_{1}^{b}\left(p,q\right)\right]=\bar{E\left[D_{2}^{w}\left(p,q\right)\right]}-E\left[D_{2}^{b}\left(p,q\right)\right]-\frac{1}{2}\|\left(p-q\right)-\left(\tilde{p}-\tilde{q}\right){\|}^{2}.$$

The extent to which $\bar{E\left[D_{1}^{w}\left(p,q\right)\right]}<E\left[D_{1}^{b}\left(p,q\right)\right]$ for **p** ≠ **q**, or $\bar{E\left[D_{1}^{w}\left(p,q\right)\right]}-E\left[D_{1}^{b}\left(p,q\right)\right]$ , has a greater absolute value than the corresponding extent to which $\bar{E\left[D_{2}^{w}\left(p,q\right)\right]}<E\left[D_{2}^{b}\left(p,q\right)\right]$ for **p** ≠ **q**, or $\bar{E\left[D_{2}^{w}\left(p,q\right)\right]}-E\left[D_{2}^{b}\left(p,q\right)\right]$ .

## 7 Data analysis

### 7.1 Data

Our theoretical analysis predicts features of dissimilarities $D_{1}$ and $D_{2}$ in within-population and between-population computations. To compare to empirical observations, we examine multiallelic microsatellite data from the Human Genome Diversity Project (HGDP-CEPH panel). We consider the 1048 individuals and 783 microsatellite loci from Rosenberg et al. (2005), employing the H1048 subset of the HGDP-CEPH panel (Rosenberg 2006). We follow previous uses of the HGDP-CEPH panel in considering 53 populations and 7 geographic regions. We focus on 30 populations for which the number of sampled individuals is greater than 15. Across these 30 populations, the total number of individuals considered is 813.

### 7.2 Theoretical computations

For our theoretical calculations, given a population in the data set and a locus, we compute allele frequencies. We then apply our theoretical formulas to the allele frequency vectors. Note that if a locus is missing genotypes in an individual, then we omit that individual from the calculation of population allele frequencies at the locus, so that we maintain the property that allele frequencies at a locus in a population sum to 1.

### 7.3 Empirical computations

For empirical calculations, we consider the actual diploid individuals in the HGDP-CEPH data, for within-population computations comparing all pairs of individuals within a population. For between-population computations, we compare all pairs of individuals, one each from two populations. Pairwise dissimilarities between diploid genotypes are obtained according to Table 1. We compute within-population and between-population dissimilarities as the means across relevant pairs, and we compute variances of dissimilarity distributions across pairs of individuals.

For this analysis, we omit individuals with missing data prior to computation of empirical ASD values. In between-population comparisons, all allelic types present in one but not the other population are assigned a frequency of 0 in the population in which they are absent.

We perform the theoretical and empirical calculations for all 783 loci.

### 7.4 Results of data analysis

**Figure 6:**
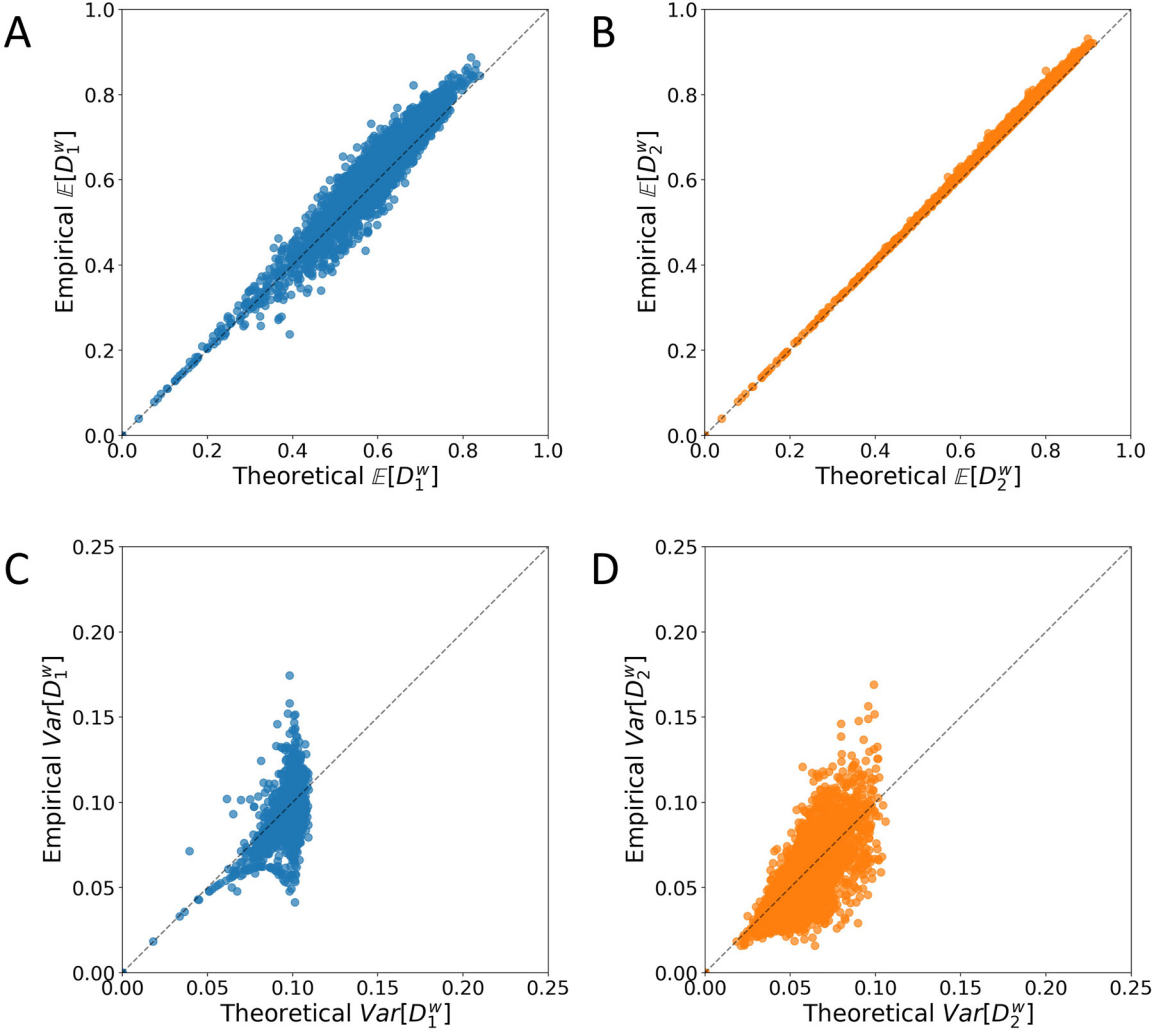
Empirical and theoretical mean and variance of within-population allele-sharing dissimilarities. Each panel considers 100 randomly sampled loci (among 783) in 30 populations with sample size greater than 15 (100 × 30 = 3000 data points in each panel). (A) $E\left[D_{1}^{w}\right]$ . (B) $E\left[D_{2}^{w}\right]$ . (C) $Var\left[D_{1}^{w}\right]$ . (D) $Var\left[D_{2}^{w}\right]$ . Empirical values rely on dissimilarity calculations according to Table 1 from pairs of diploid individuals, and theoretical values are calculated from allele frequencies according to Eqs. (3), (6), (9) and (12).

Figure 6 compares empirical and theoretical means and variances of within-population dissimilarities across pairs of individuals, considering 100 randomly sampled loci in 30 populations. Figure 6A compares the empirical value of $E\left[D_{1}^{w}\right]$ computed by averaging $D_{1}^{w}$ values for all pairs of sampled individuals with the theoretical value predicted from the allele frequencies and Eq. (3). The theoretical calculation generally predicts the empirical dissimilarity, with most points clustering along the diagonal (*r* = 0.962). In Figure 6B, a similar plot for $E\left[D_{2}^{w}\right]$ using Eq. (9) for the theoretical computation produces closer agreement between the empirical and theoretical values (*r* = 0.999).

Figure 6C and D compare empirical and theoretical variances across pairs of individuals for within-population dissimilarities, using Eqs. (6) and (12) for the theoretical computation. The theoretical variance predicts the empirical variance, but the agreement is not as close as for the mean (*r* = 0.676 for $Var\left[D_{1}^{w}\right]$ , *r* = 0.732 for $Var\left[D_{2}^{w}\right]$ ).

**Figure 7:**
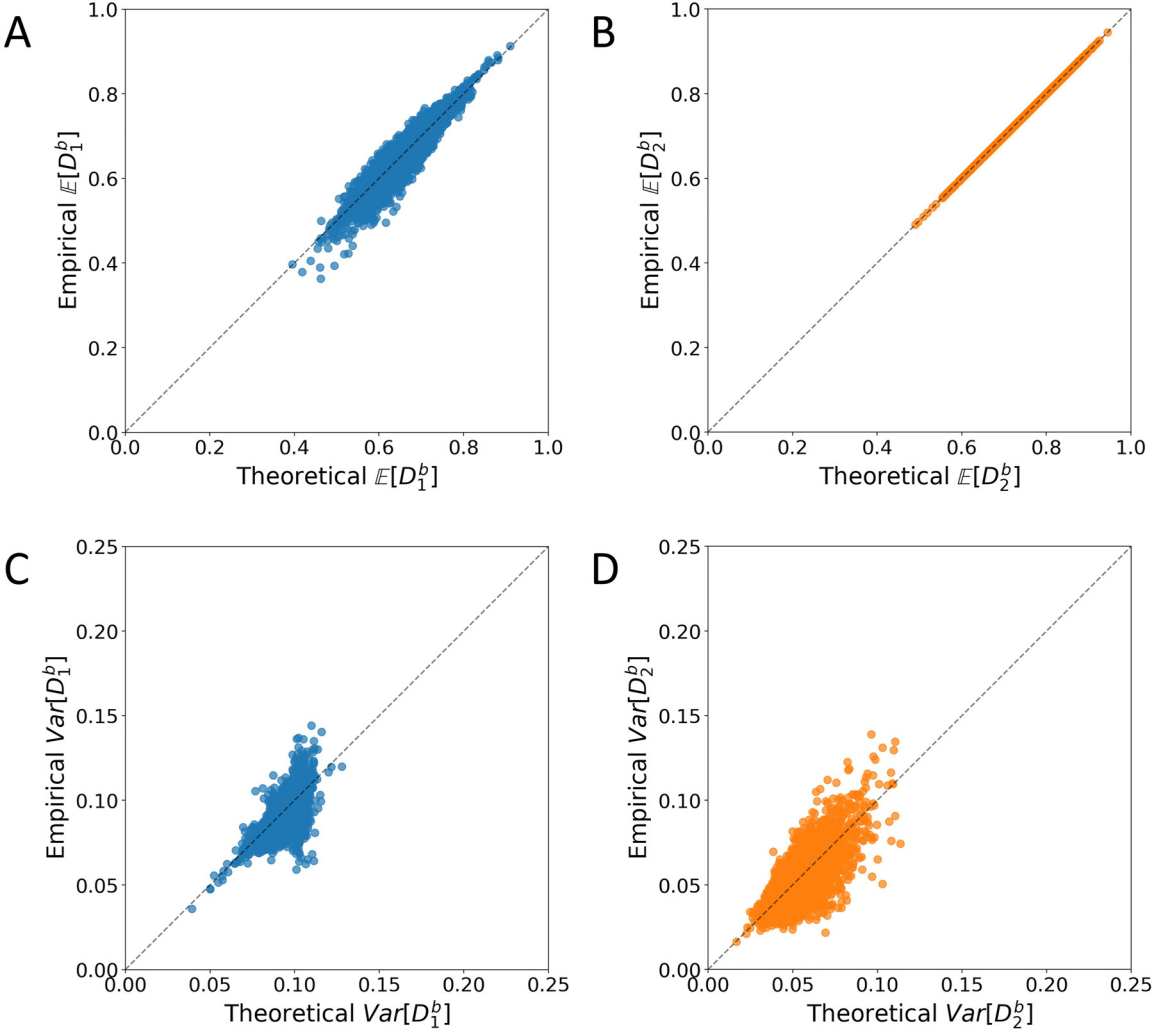
Empirical and theoretical mean and variance of between-population allele-sharing dissimilarities. Each panel considers 10 randomly sampled loci in pairs among the 30 populations with sample size greater than 15 ( 10×302=4350 data points in each panel). The 10 loci are taken from among those used in Figure 6. (A) $E\left[D_{1}^{b}\right]$ . (B) $E\left[D_{2}^{b}\right]$ . (C) $Var\left[D_{1}^{b}\right]$ . (D) $Var\left[D_{2}^{b}\right]$ . Empirical values rely on dissimilarity calculations according to Table 1 from pairs of diploid individuals, and theoretical values are calculated from allele frequencies according to Eqs. (16), (19), (22) and (25).

Figure 7 plots analogous comparisons for between-population dissimilarities, considering a subset of loci from Figure 6. In Figure 7A, we see a close relationship between empirical $E\left[D_{1}^{b}\right]$ and theoretical $E\left[D_{1}^{b}\right]$ similar to the relationship observed in Figure 6A (*r* = 0.943). As was seen in Figure 6B, in Figure 7B, we see a stronger relationship between the empirical value of $E\left[D_{2}^{b}\right]$ and the theoretical value (*r* = 1.000).

Figure 7C and D consider relationships between empirical and theoretical between-population variances for $D_{1}$ and $D_{2}$ . As was observed in Figure 6C and D, empirical and theoretical variance are correlated (*r* = 0.676 for $Var\left[D_{1}^{b}\right]$ , *r* = 0.731 for $Var\left[D_{2}^{b}\right]$ ), but the agreement for variances is not as close as for the mean.

**Figure 8:**
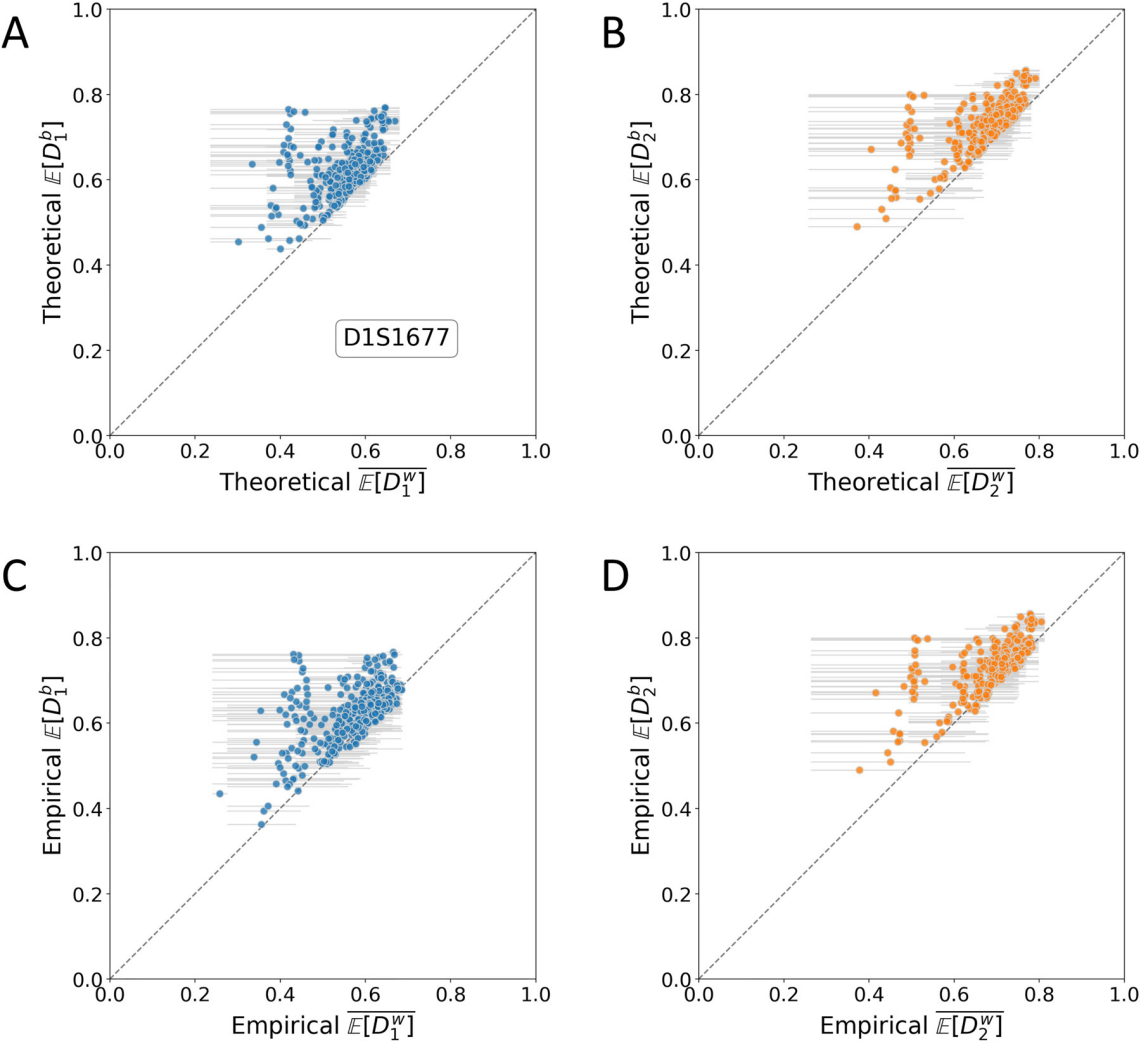
Empirical and theoretical $E\left[D^{b}\right]$ and $\bar{E\left[D^{w}\right]}$ . Each panel considers a random locus, D1S1677, in 435 pairs of populations with sample size greater than 15. The locus is among those used in Figures 6 and 7. The upper left triangle is the region in which the between-population dissimilarity of two populations exceeds the mean of the within-population dissimilarities of the two populations, $E\left[D^{b}\right]>\bar{E\left[D^{w}\right]}$ , as proven for theoretical disimilarities (Theorems 3 and 4). The two ends of a horizontal gray line indicate the $E\left[D^{w}\right]$ values for two populations whose mean within-population dissimilarity is plotted at the midpoint of the line. (A) Theoretical values of $D_{1}$ . (B) Theoretical values of $D_{2}$ . (C) Empirical values of $D_{1}$ . (D) Empirical values of $D_{2}$ .

Figure 8 empirically examines the inequalities in Theorems 3 and 4 stating that when computed from allele frequencies, the mean of the within-population dissimilarities for two populations is always less than the dissimilarity between them. It shows all population pairs from Figures 6 and 7 with a single random locus.

In Figure 8A, we find that the theoretical values of $E\left[D_{1}^{b}\right]$ and $\bar{E\left[D_{1}^{w}\right]}$ , computed from allele frequencies alone, follow the predicted inequality, with $E\left[D_{1}^{b}\right]>\bar{E\left[D_{1}^{w}\right]}$ . However, the theorem does not necessarily apply to dissimilarities computed from actual diploid individuals, and indeed, some exceptions are observed in which the empirical $E\left[D_{1}^{b}\right]$ is smaller than $\bar{E\left[D_{1}^{w}\right]}$ (Figure 8C). Similar results hold for $E\left[D_{2}^{b}\right]$ and $\bar{E\left[D_{2}^{w}\right]}$ inFigure 8B and D.

**Figure 9:**
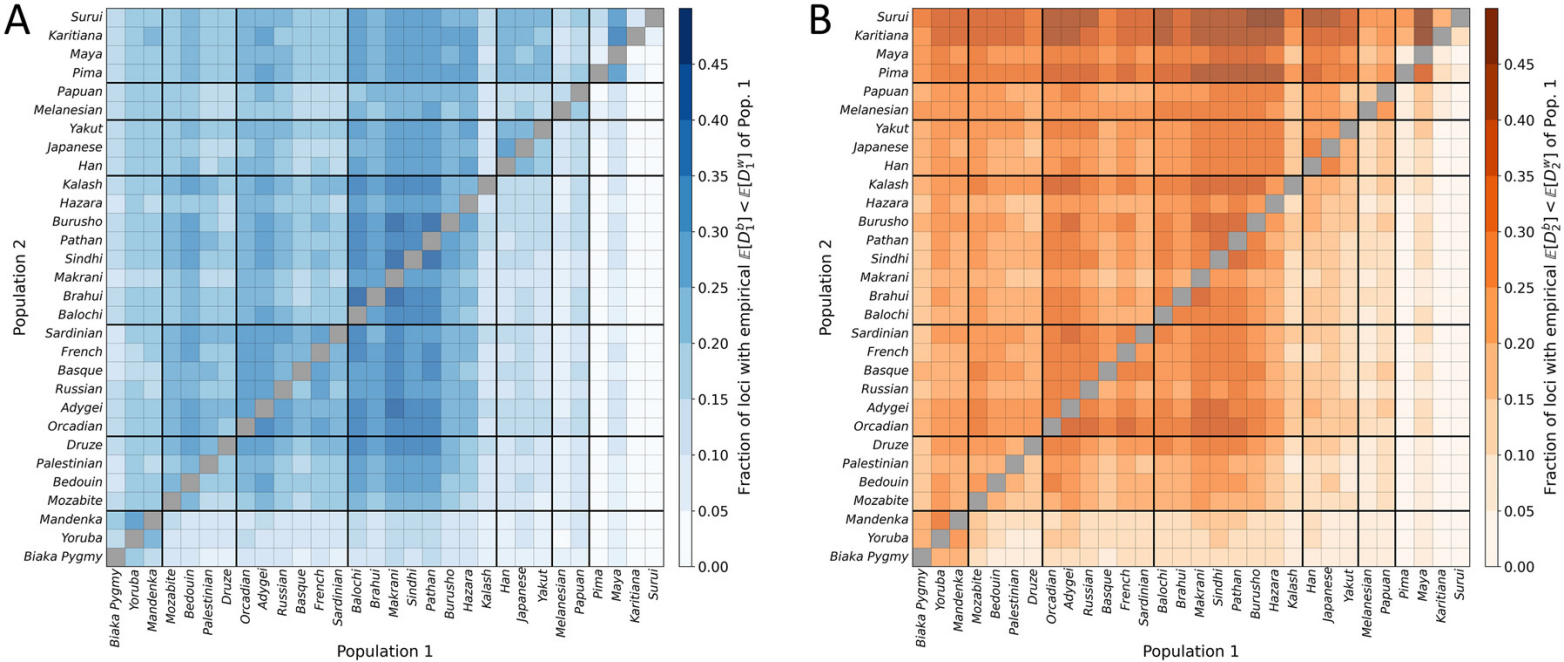
Fraction of loci for which $E\left[D^{b}\right]<E\left[D^{w}\right]$ . Each panel considers all 783 loci in pairs among the 30 populations with sample size greater than 15. Each cell denotes a pair of populations, with Population 1 considered for the within-population dissimilarity. Geographical regions are separated by bold black lines. (A) $D_{1}$ . (B) $D_{2}$ .

Figure 9 tabulates the fraction of loci for which the empirical within-population dissimilarity of a population (denoted Population 1) exceeds the population’s empirical between-population dissimilarity with a second population (Population 2), or $E\left[D^{w}\right]>E\left[D^{b}\right]$ . The populations are arranged geographically, following a general decrease in within-population genetic diversity with migration distance from Africa, as measured by expected heterozygosity 1 − *σ*_2_ (Prugnolle et al. 2005; Ramachandran et al. 2005). In Figure 9A, for $D_{1}$ , if Population 1 is a population with relatively low within-population heterozygosity, such as a Native American population, then its within-population dissimilarity rarely exceeds its between-population dissimilarity with a second population (rightmost columns). The fraction of loci for which $E\left[D^{w}\right]>E\left[D^{b}\right]$ is greatest for intermediate-heterozygosity South Asian populations (central columns). If Population 2 is a high-heterozygosity African population, then for all non-African choices of Population 1, the within-population dissimilarity of Population 1 rarely exceeds the between-population dissimilarity with an African Population 2 (bottom rows). Similar patterns are seen in Figure 9B for $D_{2}$ , with the additional observation that the within-population dissimilarity of Population 1 often exceeds the between-population dissimilarity when low-heterozygosity Native American populations are placed in the role of Population 2 (top rows).

## 8 Discussion

Allele-sharing statistics are often used to quantify genetic dissimilarity within and between populations. Because they typically share a larger number of recent ancestors, individuals from the same population might be predicted to possess a lower genetic dissimilarity than those from different populations. We have mathematically explored the circumstances under which this prediction fails, when the genetic dissimilarity within a population exceeds the genetic dissimilarity between two populations. The analysis characterizes the properties of allele frequency vectors that give rise to this counterintuitive scenario, illustrating its occurrence in human population-genetic data.

When does within-population dissimilarity for a population exceed between-population dissimilarity with a second population? The conditions that permit this inequality in the case of *I* = 2 alleles are instructive (Theorems 1 and 2 and Figure 4). In this case, two populations have unbalanced allele frequencies, with Population 2 more unbalanced than Population 1, but the two populations are similar in their frequencies. In Population 1, dissimilarity is generated from comparisons of homozygotes for one allele and homozygotes for the other allele. However, because Population 2 has allele frequencies that are more unbalanced than those of Population 1, fewer comparisons of distinct homozygotes occur in the between-population comparison. This phenomenon results in a within-population dissimilarity in Population 1 that exceeds the between-population dissimilarity. Beyond *I* = 2, such an excess is observed in empirical calculations with *I* ≥ 2 alleles (Figure 9), as well as in simulations, though with decreasing probability as *I* increases (Figure 5).

Although a population can possess greater within-population dissimilarity than its between-population dissimilarity to a second population, we find that for arbitrary numbers of alleles *I*, it is not possible for *both* populations in a pair to possess greater within-population dissimilarity than the between-population dissimilarity (Theorems 3 and 4). In data, “theoretical” dissimilarities obtained by treating allele frequencies in the data as parametric frequencies of two populations follow this inequality strictly, with greater between-population dissimilarity than at least one of the two within-population dissimilarities (Figure 8A and B). Similarly, the mean of the two within-population dissimilarities is strictly less than the between-population dissimilarity in theoretical calculations (Figure 8A and B); while “empirical” dissimilarities calculated from individual genotypes *can* violate the inequality, we find that these violations are generally mild (Figure 8C and D).

The results can contribute to understanding unexpected phenomena involving allele-sharing dissimilarities in human populations. We have seen that within-population dissimilarities in Population 1 sometimes exceed between-population dissimilarities, often in comparisons that involve a lower-diversity Population 2 and a higher-diversity Population 1 (Figure 9); in essence, a high-diversity population can possess enough variation that its inter-individual dissimilarity can exceed the dissimilarity between populations. Our theoretical calculations provide a basis for this scenario, and in fact, we saw for *I* = 2 that it is not unlikely in certain parts of the allele frequency space (Figure 4).

Our theoretical analysis deepens a line of inquiry on mathematical effects on allele-sharing. For each of two dissimilarity functions, we have obtained probability distributions of within- and between-population allele-sharing dissimilarities across pairs of individuals as functions of allele frequencies (Tables 3, 4, 6, 7), focusing on the mean and variance of the dissimilarity statistics (Eqs. (3), (6), (9), (12), (16), (19), (22) and (25)). The expressions for these quantities, and inequalities concerning their relationships (Theorems 1–4), augment previous efforts on the mathematics of allele-sharing dissimilarities in terms of allele frequencies (Chakraborty and Jin 1993; Tal 2013).

The two variants of allele-sharing dissimilarity that we studied, $D_{1}$ and $D_{2}$ , share many features. For *I* = 2 and *I* = 3 alleles, the expected values of $D_{1}^{w}$ and $D_{2}^{w}$ are maximal when all alleles have the same frequency (Figures 1A and 2A, B). Trends in expectations of $D_{1}^{b}$ and $D_{2}^{b}$ at *I* = 2 are also similar (Figure 3A and B), as are the regions in which $E\left[D^{w}\right]>E\left[D^{b}\right]$ for *I* = 2 (Figure 4), and the simulated probabilities $P\left(E\left[D^{w}\right]>E\left[D^{b}\right]\right)$ for *I* ≥ 2 (Figure 5).

However, some consistent differences between the two dissimilarities are also observed. $D_{2}\geq D_{1}$ for all genotypes (Table 1), and hence, $E\left[D_{2}^{w}\right]\geq E\left[D_{1}^{w}\right]$ (Figures 1 and 2C and Eq. (15)) and $E\left[D_{2}^{b}\right]\geq E\left[D_{1}^{b}\right]$ (Figure 3C and Eq. (28)). Although both dissimilarities have $\bar{E\left[D^{w}\right]}\leq E\left[D^{b}\right]$ (Theorems 3 and 4), $\bar{E\left[D_{1}^{w}\right]}-E\left[D_{1}^{b}\right]\leq \bar{E\left[D_{2}^{w}\right]}-E\left[D_{2}^{b}\right]$ (Eq. (39)), so that the extent to which $\bar{E\left[D^{w}\right]}$ lies below $E\left[D^{b}\right]$ has greater magnitude for $D_{1}$ .

The within-population variance across pairs of individuals is not uniformly higher for either dissimilarity (Figures 1B and 2F); at *I* = 2, it has different shapes, as $Var\left[D_{2}^{w}\right]$ has two maxima, whereas $Var\left[D_{1}^{w}\right]$ has only one (Figure 1B). $D_{2}$ has larger regions in which $E\left[D^{w}\right]>E\left[D^{b}\right]$ for *I* = 2 (Figure 4) and for *I* ≥ 2 (Figure 5). In the empirical analysis, $D_{2}$ has a closer match between empirical and theoretical mean values of the dissimilarity (Figures 6B and 7B). Its patterns in the fraction of loci for which $E\left[D^{w}\right]>E\left[D^{b}\right]$ align more closely with the heterozygosity values of the populations, with the probability of $E\left[D^{w}\right]>E\left[D^{b}\right]$ larger when Population 1 is a higher-diversity population and Population 2 is a lower-diversity population (Figure 9B). Notably, expressions for $E\left[D_{2}\right]$ are closely tied to heterozygosity (Eq. (9)) and its between-population analogue (Eq. (22)), potentially explaining the tighter connection of heterozygosity to its associated observations. Thus, the lesser-used $D_{2}$ – which, unlike $D_{1}$ , allows the dissimilarity of an individual and itself to be nonzero (Table 1) – does possess a more easily interpreted pattern in the probability that $E\left[D^{w}\right]>E\left[D^{b}\right]$ .

Does our analysis suggest a preference for $D_{1}$ over $D_{2}$ , or vice versa? To summarize, $D_{1}$ has been used more frequently than $D_{2}$ , and it also has the property that the dissimilarity of an individual and itself is zero. The less frequently used $D_{2}$ does not have this property, but it produces simpler expressions for its within-population and between-population expectations, with more natural interpretations of those expectations and their consequences. We conclude that although $D_{1}$ has a number of desirable properties, $D_{2}$ does as well, and it perhaps merits attention commensurate with that given to $D_{1}$ .

This work has several possible extensions. We have focused on the first and second moments of allele-sharing dissimilarities across pairs of individuals; the full distributions (Tables 3, 4, 6, 7) could also be further investigated. We examined *I* = 2 in the greatest detail, but special cases that fix a maximal value of *I* could also be considered. We chose the two most frequently used ASD variants, $D_{1}$ and $D_{2}$ , but a variant designed for genotypes obtained by observation of band patterns (Chakraborty and Jin 1993) could also be studied.

We have only considered allele-sharing dissimilarity between population pairs at a single locus, and it will be of interest to investigate dissimilarities that average across many loci. Our theoretical calculations focus on dissimilarities between two random individuals chosen from specified allele-frequency distributions at a locus. Although such distributions have nonzero probability only on the discrete values $\left\{0,\frac{1}{2},1\right\}$ for $D_{1}$ and $\left\{0,\frac{1}{2},\frac{3}{4},1\right\}$ for $D_{2}$ , when an allele-sharing dissimilarity is calculated as an average across *L* loci, the 2*L* + 1 values $\left\{0,\frac{1}{2L},\frac{1}{L},\frac{3}{2L},\ldots ,\frac{L-1}{L},\frac{2L-1}{2L},1\right\}$ become possible values for $D_{1}$ (all multiples of $\frac{1}{2L}$ in [0,1]), and the 4*L* values $\left\{0,\frac{1}{2L},\frac{3}{4L},\ldots ,\frac{4L-3}{4L},\frac{2L-1}{2L},\frac{4L-1}{4L},1\right\}$ for $D_{2}$ (all multiples of $\frac{1}{4L}$ in [0,1], other than $\frac{1}{4L}$ itself). Thus, the mean allele-sharing dissimilarity of a random pair of individuals across many loci – computed either theoretically or empirically – has many possible numerical values, potentially giving rise to continuous approximations for associated probability distributions.

We note significant caveats in interpreting our empirical analysis in relation to our theoretical computations. The empirical computations make use of all pairs of individuals drawn from specified samples; each sampled individual appears in many pairs, so that the empirical analysis does not follow the assumption of the theoretical analysis that pairs represent independent draws from allele frequency distributions. A second difference of the empirical and theoretical analyses is that the theoretical analysis assumes that pairs of alleles *within* an individual are independent draws from the allele-frequency distribution, whereas inbreeding can induce dependence of these alleles empirically. Such deviations from the assumptions of the theoretical analysis in conducting the empirical analysis could be explored in simulations that do and do not permit inbreeding and reuse of pairs of individuals and in empirical samples large enough to avoid such reuses.

Allele-sharing dissimilarities have long been used in population genetics. The mathematical relationships we have obtained assist both in predicting their properties in relation to allele frequencies and in understanding empirical aspects of their values. When counterintuitive phenomena are obtained with such dissimilarities – such as a greater within-population dissimilarity than the between-population dissimilarity – the mathematical results can potentially provide insight into the unexpected observations.

## References

[j_sagmb-2023-0004_ref_001] Bowcock A.M., Ruiz-Linares A., Tomfohrde J., Minch E., Kidd J.R., Cavalli-Sforza L.L. (1994). High resolution of human evolutionary trees with polymorphic microsatellites. *Nature*.

[j_sagmb-2023-0004_ref_002] Cavalli-Sforza L.L., Edwards A.W.F. (1967). Phylogenetic analysis: models and estimation procedures. *Am. J. Hum. Genet.*.

[j_sagmb-2023-0004_ref_003] Chakraborty R., Jin L., Pena S.D.J., Chakraborty R., Epplen J.T., Jeffreys A.J. (1993). A unified approach to study hypervariable polymorphisms: statistical considerations of determining relatedness and population distances. *DNA fingerprinting: state of the science*.

[j_sagmb-2023-0004_ref_004] Edge M.D., Ramachandran S., Rosenberg N.A. (2022). Celebrating 50 years since Lewontin’s apportionment of human diversity. *Phil. Trans. Roy. Soc. Lond. B Biol. Sci.*.

[j_sagmb-2023-0004_ref_005] Gao X., Martin E.R. (2009). Using allele sharing distance for detecting human population stratification. *Hum. Hered.*.

[j_sagmb-2023-0004_ref_006] Jorde L.B. (1985). Human genetic distance studies: present status and future prospects. *Annu. Rev. Anthropol.*.

[j_sagmb-2023-0004_ref_007] Lewontin R.C. (1972). The apportionment of human diversity. *Evol. Biol.*.

[j_sagmb-2023-0004_ref_008] Mountain J.L., Cavalli-Sforza L.L. (1997). Multilocus genotypes, a tree of individuals, and human evolutionary history. *Am. J. Hum. Genet.*.

[j_sagmb-2023-0004_ref_009] Mountain J.L., Ramakrishnan U. (2005). Impact of human population history on distributions of individual-level genetic distance. *Hum. Genom.*.

[j_sagmb-2023-0004_ref_010] Nei M. (1972). Genetic distance between populations. *Am. Nat.*.

[j_sagmb-2023-0004_ref_011] Nei M. (1987). *Molecular evolutionary genetics*.

[j_sagmb-2023-0004_ref_012] Prugnolle F., Manica A., Balloux F. (2005). Geography predicts neutral genetic diversity of human populations. *Curr. Biol.*.

[j_sagmb-2023-0004_ref_013] Ramachandran S., Deshpande O., Roseman C.C., Rosenberg N.A., Feldman M.W., Cavalli-Sforza L.L. (2005). Support from the relationship of genetic and geographic distance in human populations for a serial founder effect originating in Africa. *Proc. Natl. Acad. Sci. USA*.

[j_sagmb-2023-0004_ref_014] Rosenberg N.A. (2006). Standardized subsets of the HGDP-CEPH human genome diversity cell line panel, accounting for atypical and duplicated samples and pairs of close relatives. *Ann. Hum. Genet.*.

[j_sagmb-2023-0004_ref_015] Rosenberg N.A. (2011). A population-genetic perspective on the similarities and differences among worldwide human populations. *Hum. Biol.*.

[j_sagmb-2023-0004_ref_016] Rosenberg N.A., Mahajan S., Ramachandran S., Zhao C., Pritchard J.K., Feldman M.W. (2005). Clines, clusters, and the effect of study design on the inference of human population structure. *PLoS Genet.*.

[j_sagmb-2023-0004_ref_017] Tal O. (2013). Two complementary perspectives on inter-individual genetic distance. *Biosystems*.

[j_sagmb-2023-0004_ref_018] Witherspoon D.J., Wooding S., Rogers A.R., Marchani E.E., Watkins W.S., Batzer M.A., Jorde L.B. (2007). Genetic similarities within and between human populations. *Genetics*.

